# Striatal Dopamine Loss in Early Parkinson's Disease: Systematic Review and Novel Analysis of Dopamine Transporter Imaging

**DOI:** 10.1002/mdc3.13687

**Published:** 2023-02-17

**Authors:** Nicholas Heng, Naveed Malek, Michael A. Lawton, Anahita Nodehi, Vanessa Pitz, Katherine A. Grosset, Yoav Ben‐Shlomo, Donald G. Grosset

**Affiliations:** ^1^ School of Neuroscience and Psychology University of Glasgow Glasgow Scotland UK; ^2^ Department of Neurology Queen's Hospital Romford Essex UK; ^3^ Population Health Sciences, Bristol Medical School University of Bristol Bristol UK

**Keywords:** Parkinson's disease, neuroimaging, dopamine transporter.

## Abstract

**Background:**

Neuropathological studies, based on small samples, suggest that symptoms of Parkinson's disease (PD) emerge when dopamine/nigrostriatal loss is around 50–80%. Functional neuroimaging can be applied in larger numbers during life, which allows analysis of the extent of dopamine loss more directly.

**Objective:**

To quantify dopamine transporter (DaT) activity by neuroimaging in early PD.

**Methods:**

Systematic review and novel analysis of DaT imaging studies in early PD.

**Results:**

In our systematic review, in 423 unique cases from 27 studies with disease duration of less than 6 years, mean age 58.0 (SD 11.5) years, and mean disease duration 1.8 (SD 1.2) years, striatal loss was 43.5% (95% CI 41.6, 45.4) contralaterally, and 36.0% (95% CI 33.6, 38.3) ipsilaterally. For unilateral PD, in 436 unique cases, mean age 57.5 (SD 10.2) years, and mean disease duration 1.8 (SD 1.4) years, striatal loss was 40.6% (95% CI 38.8, 42.4) contralaterally, and 31.6% (95% CI 29.4, 33.8) ipsilaterally. In our novel analysis of the Parkinson's Progressive Marker Initiative study, 413 cases had 1436 scans performed. For a disease duration of less than 1 year, age was 61.8 (SD 9.8) years, and striatal loss was 51.2% (95% CI 49.1, 53.3) contralaterally and 39.5% (36.9, 42.1) ipsilaterally, giving an overall striatal loss of 45.3% (43.0, 47.6).

**Conclusions:**

Loss of striatal DaT activity in early PD is less at 35–45%, rather than the 50–80% striatal dopamine loss estimated to be present at the time of symptom onset, based on backwards extrapolation from autopsy studies.

Nigrostriatal dopaminergic cell loss in Parkinson's disease (PD) was originally assessed in autopsy studies.^1^, ^2^ Neuronal loss was greatest in the ventral tier of the substantia nigra (71–91%) and least in the dorsal tier (46–48%).^2^ It was estimated from autopsy studies that 70–80% of striatal dopamine was already depleted by the time motor symptoms emerged,^1^, ^2^, ^3^ but it has subsequently been argued that this is likely to be an overestimate, given the limited availability of early‐stage cases, selection bias, effects of tissue degradation after death, and other confounding factors.^4^

Functional imaging studies allow for the measurement of presynaptic dopaminergic cell function during life, in larger numbers of cases, earlier in the disease process, and repeatedly, which increases precision in quantifying dopaminergic activity in early PD. However, individual studies are generally small, usually single center, and have variation in patient selection, the extent of clinical data collected, and the imaging radiotracer used and method of analysis, all of which influence comparisons between studies, and limit generalizability of results.

We used two approaches to address these issues. Firstly, we conducted a novel systematic review to assess DaT imaging during the first 6 years after PD diagnosis, to reach a more definitive estimate of the extent of dopamine loss than is possible from smaller individual studies. Secondly, we analyzed imaging results from a large multi‐center prospective study in early PD, that followed a standardized protocol with detailed clinical information over the first 6 years after diagnosis of PD, to examine the extent to which these findings matched those from the systematic review. We focused on findings in relation to duration from diagnosis, and to clinical staging of disease severity. We therefore aimed to synthesize available evidence and the new analysis, to define the extent of dopaminergic loss in the early stages of PD.

## Methods

### Systematic Review of Presynaptic DaT Imaging Studies in Early PD

**FIG. 1:**
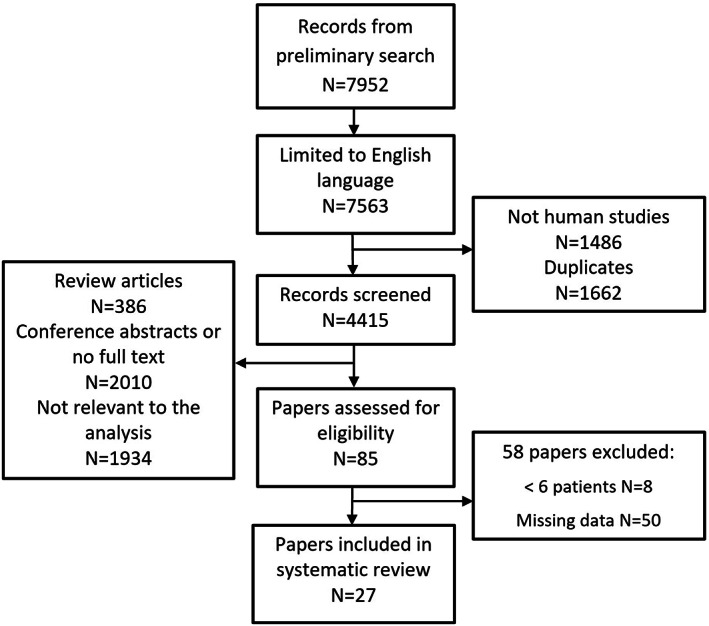
PRISMA diagram of paper selection for the systematic review.

PUBMED, EMBASE and Cochrane Library were searched for papers published between January 1, 1995 and January 31, 2021, combining the search terms: “Parkinson's disease,” “SPECT” and “PET”. Inclusion criteria required (1) a case control design, (2) >5 PD cases diagnosed using standard clinical criteria, (3) studies where disease duration was less than 6 years, and/or where data could be extracted from early unilateral PD cases, based on Hoehn and Yahr (H&Y) severity grade of either 1 (unilateral disease) or 1.5 (unilateral and axial disease) and (4) results from DaT imaging that included uptake ratios measured in one or more of striatum, putamen or caudate. When the same cases were reported in more than one paper, we chose the paper with the larger sample size. Conference abstracts, book chapters, single case reports and review papers were excluded (Fig. 1).

The methodological quality of each included study was assessed using the Quality Assessment of Diagnostic Accuracy Studies 2 tool (QUADAS‐2), which comprises four key domains: patient selection, index test, reference standard and flow and timing. Two reviewers (NH and NM) carried out independent assessment of the studies included, blinded to each other's scores. Any disagreements were resolved by discussion, or if necessary, by consultation with a third author (KG). These scores were used to summarize the risk of bias, as well as any concerns raised regarding applicability (Supplementary Table S1).

Using the brain region summary statistics for the PD cases and controls within each study, we calculated the percentage loss for the PD cases relative to controls. Standard errors for the percentage difference were derived using the delta method. We then carried out a meta‐analysis and displayed all the results in forest plots. In our forest plots we have reported both an inverse‐variance (IV) weighted fixed effect analysis and a DerSimonian‐Laird (DL) random effects analysis.^5^ We also display the I‐squared statistic as a measure of heterogeneity.^6^ An assessment for bias that might result in over‐ or under‐estimation of the degree of dopaminergic loss from smaller studies was performed using funnel plots. All analyses were carried out in STATA16 using the metan command.

### Clinical and Imaging Findings in Early PD

Data from the Parkinson's Progression Markers Initiative (PPMI) study were downloaded in February 2021 (ppmi-info.org).^7^ PD patients were recruited with a recent clinical diagnosis, with a minimum of asymmetrical rest tremor and either bradykinesia or rigidity. Cases with a revised diagnosis by consensus committee were excluded (ppmi-info.org). Striatal dopamine deficiency was quantified by DaT imaging following standard methods, as a ratio of specific to non‐specific isotope uptake, combined with visual assessment, using ^123^I‐FP‐CIT and single photon emission computed tomography (SPECT).^7^ The imaging was performed at study entry and repeated 1, 2, and 4 years later. A single scan was also available for age and sex‐matched healthy controls with no first‐degree relatives’ history of idiopathic PD. The loss of dopaminergic activity for each brain region was calculated for PD cases as a percentage in relation to average control values, following previous methods.^7^ Laterality was defined in PD cases according to predominant motor signs, with the right side being arbitrarily defined as contralateral when clinical signs were symmetrical. We applied the robust standard error method to account for repeat scans in the same individual and checked this with a sensitivity analysis using the generalized estimating equations method.^8^, ^9^

## Results

### Systematic Review

**FIG. 2:**
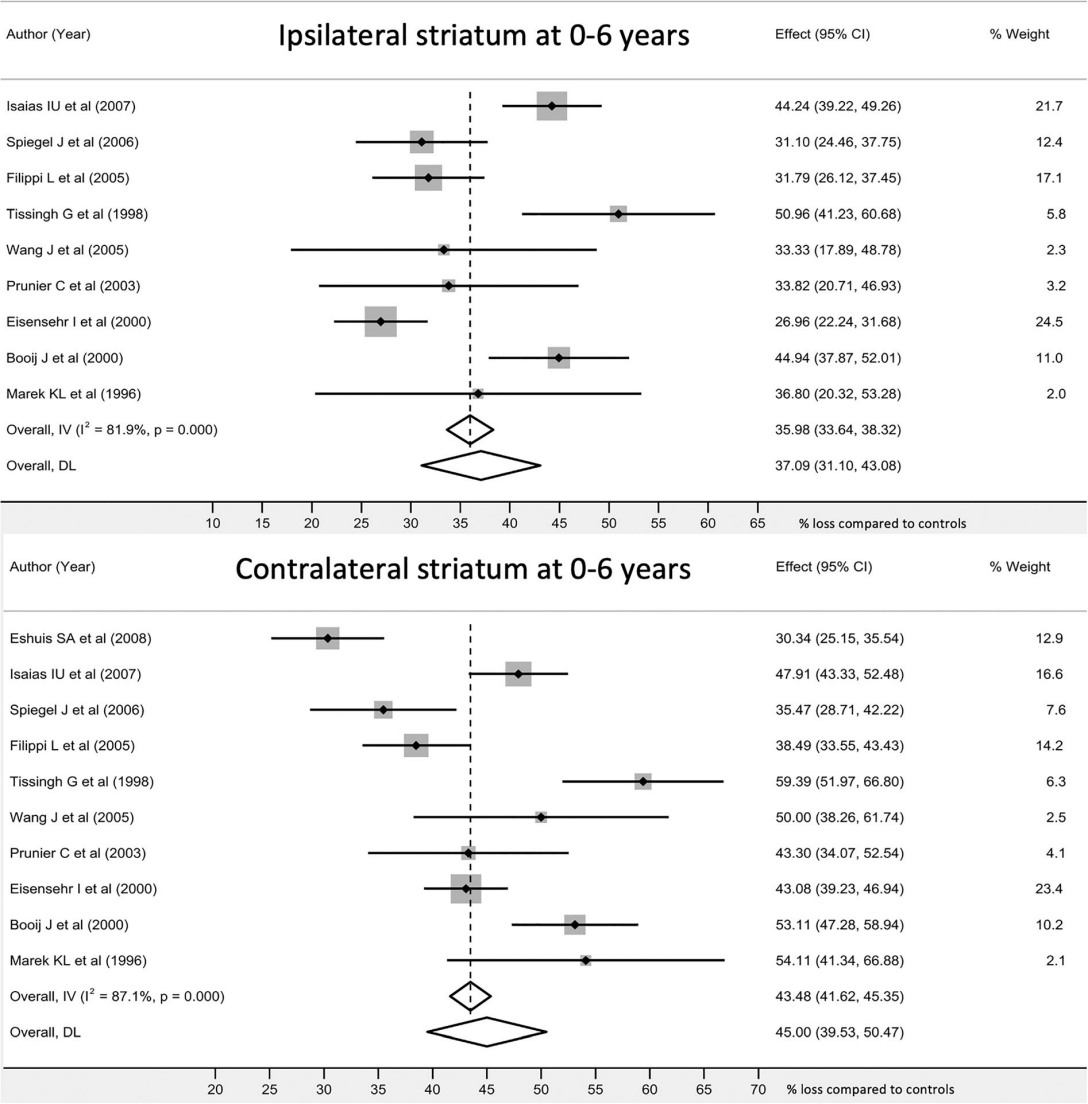
Striatal dopaminergic activity during the first six years after a diagnosis of PD. Individual and pooled analysis of studies reporting striatal values are shown. There was significant heterogeneity between studies. The overall difference between sides was 7–8%.

A total of 617 patients were identified from 27 studies (Fig. 1, Supplementary Table S2). However, some studies reported the same cases more than once, so earlier reports from the same case series were excluded, leaving 585 unique cases. For analysis of results up to 6 years after diagnosis, studies omitting disease duration were excluded; this left 423 cases from 27 studies, mean age 58.0 years (SD 11.5), 61.2% male, with mean disease duration 1.8 (SD 1.2) years. Not all studies reported findings from each striatal area (Supplementary Table S2). Where data were available, striatal loss was 36.0% (95% CI 33.6, 38.3) ipsilateral to the affected side, and 43.5% (95% CI 41.6, 45.4) contralaterally (Fig. 2); ipsilateral caudate loss was 24.2% (95% CI 22.0, 26.4); contralateral caudate loss was 31.7% (95% CI 29.8, 33.5); ipsilateral putamen loss was 42.4% (95% CI 40.6, 44.2); contralateral putamen loss was 57.9% (95% CI 56.6, 59.2) (Supplementary Fig. S1).

**FIG. 3:**
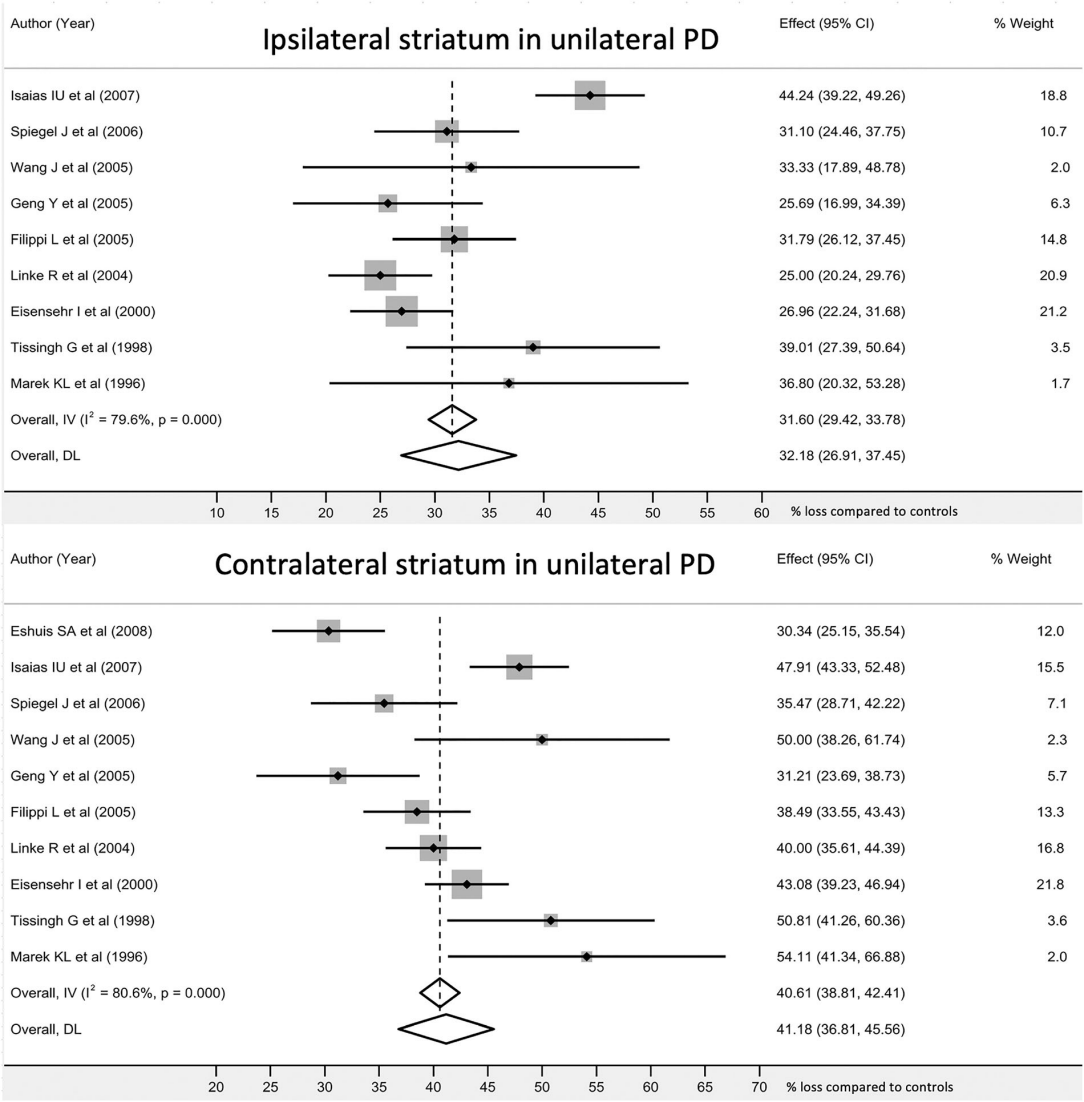
Striatal dopaminergic activity in early unilateral PD. There was significant heterogeneity between studies. There was a loss of around 32% in the clinically unaffected striatum, and the overall difference between sides was 8–9%.

Four hundred and thirty‐six unique cases (74.5% of the 585) were identified in 18 studies with unilateral disease (H&Y stage 1–1.5). Mean age was 57.5 years (SD 10.2), 58.0% were male, and the mean disease duration was 1.8 (SD 1.4) years. Using the available data for striatal areas, striatal loss was 31.6% (95% CI 29.4, 33.8) ipsilaterally and 40.6% (95% CI 38.8, 42.4) contralaterally (Fig. 3, Supplementary Table S2); ipsilateral caudate loss was 24.6% (95% CI 22.4, 26.8); contralateral caudate loss was 30.1% (95% CI 28.2, 32.0); ipsilateral putamen loss was 37.5% (95% CI 35.7, 39.3); contralateral putamen loss was 53.5% (95% CI 52.1, 54.9) (Supplementary Table S2).

In Supplementary Table S3 we displayed results for a meta‐regression of the type of tracer. Here we dichotomized tracer used into those with and without ^123^Iodine where those with ^123^Iodine group were the baseline (reference) category. In both sets of analyses (unilateral PD and 0–6 years) and for both affected sides (contralateral and ipsilateral) we have evidence that studies that used a ^123^Iodine tracer had a significantly higher percentage difference for the caudate and a significantly lower percentage difference for the putamen.

Smaller studies contributed to over‐estimating the effect size in the ipsilateral putamen for both 0–6 years (Supplementary Fig. S7, Egger test 2.62, 95% CI 1.30–3.93, *P* < 0.001) and unilateral disease (Supplementary Fig. S13, Egger test 2.37, 95% CI 0.85–3.89, *P* = 0.002). Smaller studies contributed to under‐estimating the effect size for contralateral putamen for both 0–6 years (Supplementary Fig. S8, Egger test −3.24, 95% CI −4.44 to −2.04, *P* < 0.001) and for unilateral disease (Supplementary Fig. S14, Egger test −3.50, 95% CI −4.94 to −2.07, *P* < 0.001), from assessment of the funnel plots (Supplementary Figs. S3–S8 for 0–6 years, and Supplementary Figures S9–S14 for unilateral disease).

### Novel Analysis of PPMI Study Results

Of the 423 cases recruited, six were subsequently given an alternative diagnosis, leaving 417 PD cases, of whom 413 (99.0%) had one or more DaT scan performed (Supplementary Table S4). A total of 1421 scans were performed from 0 to 6 years after diagnosis, with a further 15 scans in years 6 to 7. Two hundred and seventy‐nine scans were performed when motor features were unilateral. One hundred and ninety‐three controls (64.2% male) had a single scan at a mean age of 60.9 (SD 11.3) years, and mean uptake ratios were 2.982 (SD 0.625) for caudate and 2.147 (SD 0.555) for putamen.

#### Analysis of Early Unilateral Disease

Ninety‐five DaT scans (22.8% of 417) were performed within 1 year of diagnosis, in drug naïve cases with unilateral motor features (Supplementary Table). These cases were on average 58.2 (SD 10.4) years old at diagnosis, 60.0% were male, and the mean time from diagnosis was 0.4 (SD 0.2) years. Average striatal loss was 40.5% (95% CI 36.9–44.2), consisting of ipsilateral striatal loss at 33.7% (95% CI 29.5–37.9) and contralateral striatal loss at 47.4% (95% CI 44.0–50.8). Putamen and caudate values are in Supplementary Table S5.

#### Analysis by Duration of Diagnosis

**FIG. 4:**
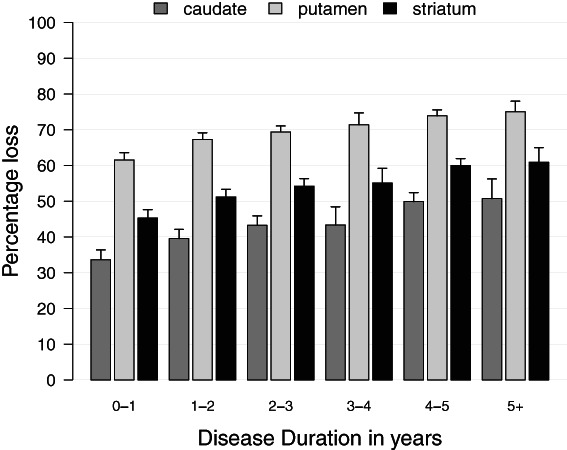
Striatal dopaminergic activity in early PD expressed as a percentage of mean values in matched controls. Striatal dopaminergic loss increased gradually, from 46.4% within 1 year of diagnosis, to 62.3% after more than 5 years. Data are mean and SD.

Three hundred and sixty‐four DaT scans (87.3% of 417) were performed within 1 year of diagnosis. These cases were on average 61.8 (SD 9.8) years old at diagnosis, 65.7% were male, at the mean time of imaging 347 (98.1% of 354) remained drug‐naïve, and the time from diagnosis was 0.4 (SD 0.2) years (Supplementary Table S4). The percentage loss increased progressively over time for all striatal areas (Fig. 4, Supplementary Table S5). Overall striatal loss was 45.3% (95% CI 43.0–47.6) within a year of diagnosis, and this increased gradually to 60.9% (95% CI 56.8–65.0) in scans performed more than 5 years after diagnosis.

Considering all DaT scans performed at 6 years or less after diagnosis, there were 1421 scans, mean age was 63.7 (SD 9.8) years, 65.2% were male, mean disease duration was 2.1 (SD 1.5) years, and overall striatal loss was 52.5% (95% CI 50.5–54.4). Lateralized and putamen and caudate values are in Supplementary Table S5.

#### Analysis by Disease Stage

**FIG. 5:**
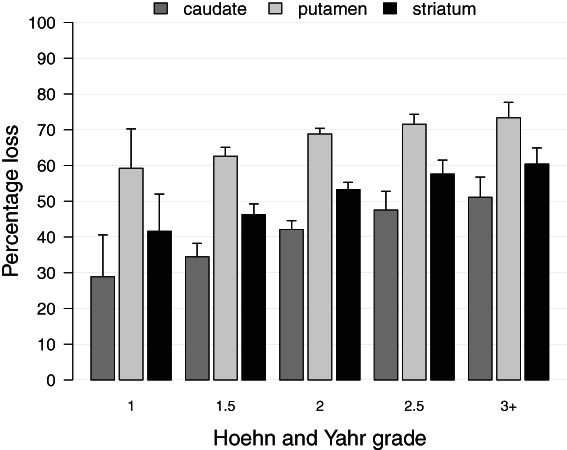
Striatal dopaminergic activity in relation to severity grade, expressed as a percentage of mean values in matched controls. For striatum, losses increased from 43.2% at Grade 1% to 62.2% at Grade 3 and higher. Data are mean and SD.

H&Y was recorded at the time of scanning for 1422 of the 1436 scans (99.0%). Motor features were unilateral in 279 (H&Y grade 1 n = 9, grade 1.5 n = 270) and bilateral in 1143 (grade 2 n = 1085, grade 3 or higher n = 58). There was a progressive increase across the H&Y stages in age, disease duration, the proportion on anti‐Parkinson's therapy and the percentage loss in each of the striatal brain areas (Fig. 5, Supplementary Tables S4 and S5).

Our primary analysis method (robust standard errors) took account of the effect of repeat scans in the same individuals; sensitivity analysis using generalized estimating equations showed virtually identical results (Supplementary Table S6).

The combined analysis from our systematic review and analysis of the PPMI cohort allows us to define the extent of dopaminergic depletion more accurately in early PD. The conclusions from early autopsy studies, that clinical signs only appeared when caudate dopamine is depleted by 70–80%^1^ or when 80% of striatal dopamine was lost^2^, ^8^ are frequently cited^8^, ^9^ but appear to be overestimates of the true situation. In this systematic review and meta‐analysis, caudate DaT activity was reduced by 25–30%, and striatal DaT activity was reduced by 35 to 45%. These findings were consistent with data from PPMI in the first year after diagnosis, where caudate DaT loss was around 35%, and striatal DaT loss was around 45%. When drug naïve cases with unilateral disease within a year of in the first diagnosis from PPMI were analyzed, average striatal DaT loss was around 40%. We therefore conclude that, for both caudate and overall striatal dopaminergic activity, the extent of dopamine loss in early disease is considerably lower than the loss at symptom onset estimated from backwards extrapolation of dopamine measurements at autopsy. There are several possible explanations for the autopsy dopamine estimates being misleading, as reviewed by Cheng et al.^4^ It is also possible that more severely affected cases were included in autopsy studies (selection bias), giving higher than average dopaminergic cell losses.

We can also compare the imaging findings with those from the other main autopsy technique of cell counts in the substantia nigra. At autopsy, cell loss at death was greatest in the lateral ventral tier of the nigra at 91%, and backwards projection suggested that there was a 64–68% cell loss in this area at the time of diagnosis.^2^ For the four cases (out of a total of 20) in that study that had a disease duration of 6 years or less, the loss in this area averaged 76%.^2^ Given that this part of the nigra projects to the putamen, our imaging analysis of the putamen is anatomically comparable. In PPMI, within the first year of diagnosis DaT loss in the putamen was around 60%. It was not possible to derive a figure within 1 year of diagnosis from the systematic review but considering scans in the first 6 years of diagnosis, DaT loss in the putamen was around 50%. Accordingly, the putamen values from DaT imaging suggest greater preservation of this part of the dopaminergic system in the early stages of PD than was calculated from backwards projections to the time of disease onset, based on cell counts at autopsy.

An additional autopsy‐to‐imaging comparison can be made for nigrostriatal neurons, comparing cells counts in the substantia nigra pars compacta with the total striatal loss on DaT imaging. For the 4 cases at autopsy with a disease duration of 6 years or less, average nigral cell loss was 49%^2^; in our systematic review striatal DaT loss was 37%, and in the PPMI cohort it was around 53%. A similar comparison can be made for later disease stages, from a combined analysis of 12 autopsy studies where cell counts were made by stereological counting, which reduces potential biases.^10^ In a total of 181 brains, the average cell loss in the substantia nigra was 68% *at the time of death* which was at a disease duration of 8 to 15 years in those studies where average disease duration was stated.^10^ This value is broadly consistent with the findings in the PPMI study, where loss of DaT activity was around 60%, both for cases with a disease duration over 5 years, and for those with a more advanced disease stage (H&Y stage 3 or higher). These figures should be considered in the context of information that rates of loss in DaT activity are reverse exponential, with a floor effect, as previously observed in the PPMI dataset.^11^

Dopamine levels were lower for all striatal areas in the PPMI study than those from our systematic review. Within the systematic review, we found clear evidence that putamen readings were lower when a ^123^Iodine label was used, compared to other radiolabels, while caudate readings were higher. This has not been reported previously to our knowledge, and should be considered in future studies, that should preferably also include striatal values.

One major difference between the systematic review and the PPMI study was the proportion of cases with unilateral disease: almost three‐quarters of cases in the systematic review compared with just under one fifth of scans in the PPMI study. Since overall striatal loss is much less in unilateral than in bilateral disease, it is not surprising that the systematic review gave lower values for overall striatal loss than was found in the PPMI study. Since there was often a specific focus on early unilateral disease in the systematic review, it was not possible to extract sufficient data for bilateral disease cases in the systematic review, to compare against the PPMI study findings. However, even for unilateral disease cases, striatal losses were higher in the PPMI study than in the systematic review. Other differences including disease duration, effects of anti‐Parkinson's therapy on disease staging, and methods to delineate the striatal brain areas, may also explain the variation in findings between the systematic review and the PPMI study. However, the much larger size and the greater number of repeated clinical and imaging observations in the PPMI cohort, in comparison to all the other studies, allow for a more detailed understanding of changes in dopamine levels in early‐stage PD.

There was clear evidence in the PPMI study that the asymptomatic hemisphere in unilateral PD is already substantially affected, which is in keeping with several studies within the review^12^, ^13^, ^14^ and is also consistent with other imaging techniques such as ^18^F‐dopa PET.^15^ In PPMI, striatal loss was 36–40% in the asymptomatic side, and this increased to around 50% loss by the time this side became symptomatic.

An important functional role of DAT is to maintain relatively constant synaptic DA levels and to preserve DA in nerve terminals.^16^ Down regulation of dopamine transporters helps to maintain synaptic cleft dopamine levels in PD,^17^ so the values from the DaT imaging studies overestimate dopaminergic cell loss. In contrast, when presynaptic dopamine imaging is performed with ^18^F dopa and other tracers that are metabolized intra‐neuronally, there is up‐regulation of aromatic acid decarboxylase in order to increase dopamine synthesis in the disease state.^17^ Such studies have generally found lower levels of dopamine loss than DaT imaging studies, being around a 33% striatal loss.^18^, ^19^, ^20^, ^21^, ^22^ We can therefore conclude that the true extent of nigrostriatal dopaminergic cell loss is likely to be between the values from each of the main imaging methods and is around a 35–45% loss within the first year after diagnosis.

Collectively, the findings of the review and novel analysis suggest that there are more surviving dopaminergic neurons available for neuroprotective treatment approaches than the earlier studies suggested. However, the disease process is already substantially progressed even within 1 year of diagnosis, even within the hemisphere corresponding to the asymptomatic side, so approaches that focus on earlier treatment in the prodromal (pre‐motor) stage of PD may have greater benefit.

Our study has certain limitations. In the systematic review, we included imaging studies of the presynaptic dopamine transporter, but did not include studies such as with ^18^F‐dopa that identify intraneuronal presynaptic dopamine activity. In the PPMI study, imaging was performed at 1, 2 and 4 years after study entry which was on average 7 months after diagnosis, and accordingly there were relatively few scans performed at a disease duration of 3 years. Finally, although there is evidence of down‐regulation of DaT in PD,^17^ we were not able to quantify the magnitude of this in relation to the current analysis.

## Conclusion

Loss of striatal DaT activity in early PD is around 35–45%, which is much lower than the back extrapolations from autopsy that estimated 50–70% nigrostriatal cell loss, and 70–80% dopamine loss, at disease onset. Since there is down‐regulation of DaT in PD, even the 35–45% range may be an overestimate. These findings are encouraging for the application of potential neuroprotective agents, at the stage of early motor presentation.

## Author Roles

(1) Research project: A. Conception and planning of project, B. Electronic search, C. Literature review; (2) Statistical analysis: A. Data analysis, B. Data interpretation; (3) Manuscript: A. Writing, B. Editing, C. Reviewing.

N.H.: 1B, 1C, 2A, 3A, 3B.

M.L.: 1A, 2A, 2B, 3B, 3C.

A.N.: 2A, 2B.

N.M.: 1B, 1C, 3A, 3B.

V.P.: 2A, 3B.

K.G.: 1A, 2A, 2B, 3A, 3B, 3C.

Y.B.S.: 1A, 2A, 2B, 3B, 3C.

D.G.: 1A, 2A, 2B, 3A, 3B.

## Disclosures

**Ethical Compliance Statement:** The authors confirm that the approval of an institutional review board and informed patient consent was not required for this work. We confirm that we have read the Journal's position on issues involved in ethical publication and affirm that this work is consistent with those guidelines.

**Funding Sources and Conflicts of Interest:** NH, ML, NM, VP, AN, KG, YBS report no conflicts of interest. DG: has received honoraria from AbbVie, Bial Pharma, Merz Pharma, GE Healthcare, and consultancy fees from the Glasgow Memory Clinic.

**Financial Disclosures for the Previous 12 Months:** The authors declare that there are no additional disclosures to report.

## Supporting information


**Figure S1.** Caudate and putamen dopaminergic activity at 0 to 6 years post‐diagnosis. There was a loss of around 24% in the clinically unaffected caudate, and around 42% in the clinically unaffected putamen. Contralateral losses were around 32% for caudate and 58% for putamen.

**Figure S2.** Caudate and putamen dopaminergic activity in early unilateral PD. Loss was around 25% in the clinically unaffected caudate, and around 38% in the clinically unaffected putamen. The contralateral caudate loss was around 30% loss.

**Figures S3–S8.** Funnel plots of studies at 0–6 years after diagnosis, assessing for publication and selection bias. The largely symmetrical pattern around the midline vertical in study position (each study represented by one dot) showed no evidence of a systematic bias.

**Figures S9–S14.** Funnel plots of studies of unilateral PD, assessing for publication and selection bias. The largely symmetrical pattern around the midline vertical in study position (each study represented by one dot) showed no evidence of a systematic bias.

**Table S1.** Risk bias analysis of papers based on the QUADAS‐2 tool evaluating the diagnostic use of dopamine transporter imaging in early Parkinson's disease.
**Table S2.** Summary of 27 case control studies evaluating the diagnostic use of dopamine transporter imaging in early Parkinson's disease.
**Table S3.** Meta‐regression for type of tracer used. Reference (baseline) category is an isotope with ^123^Iodine
**Table S4.** Patient characteristics in the PPMI study.
**Table S5.** Striatal dopamine loss by disease duration and grading in the PPMI study, analyzed by robust standard errors.
**Table S6.** Striatal dopamine loss by disease duration and grading in the PPMI study, analyzed using generalized estimating equations.
