# Supplementary figures and images for: Striatal Dopamine Loss in Early Parkinson's Disease: Systematic Review and Novel Analysis of Dopamine Transporter Imaging
Source: Mov Disord Clin Pract. 2023 Feb 17;10(4):539–46. doi: 10.1002/mdc3.13687 (PMC10105104; doi:10.1002/mdc3.13687)

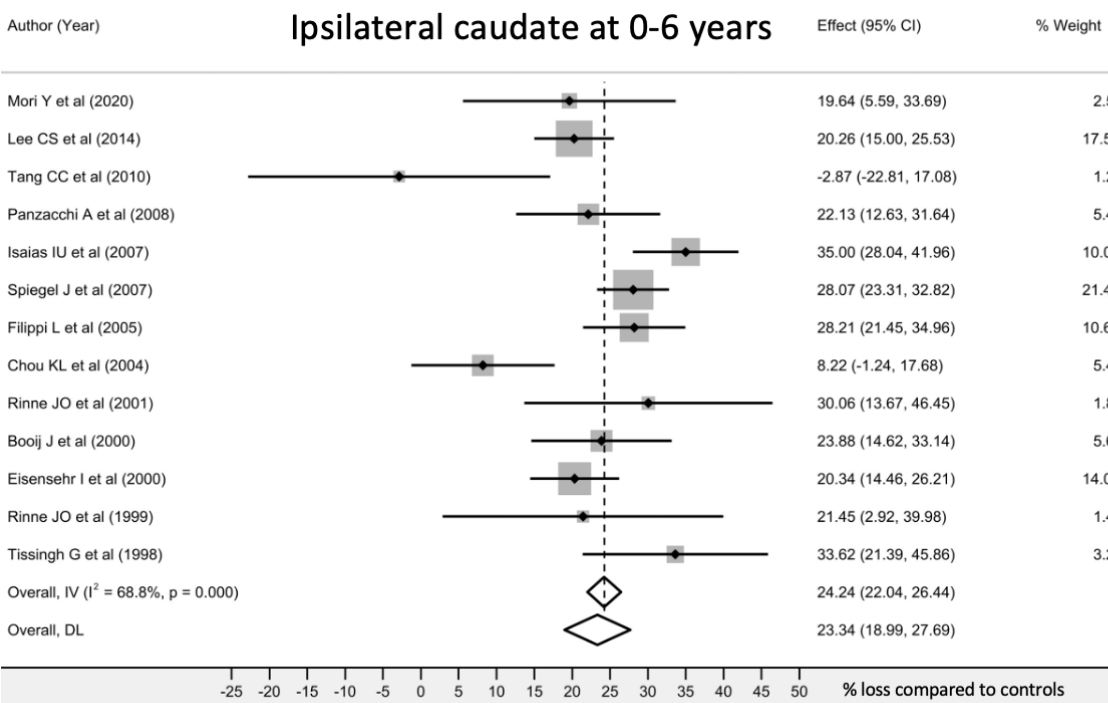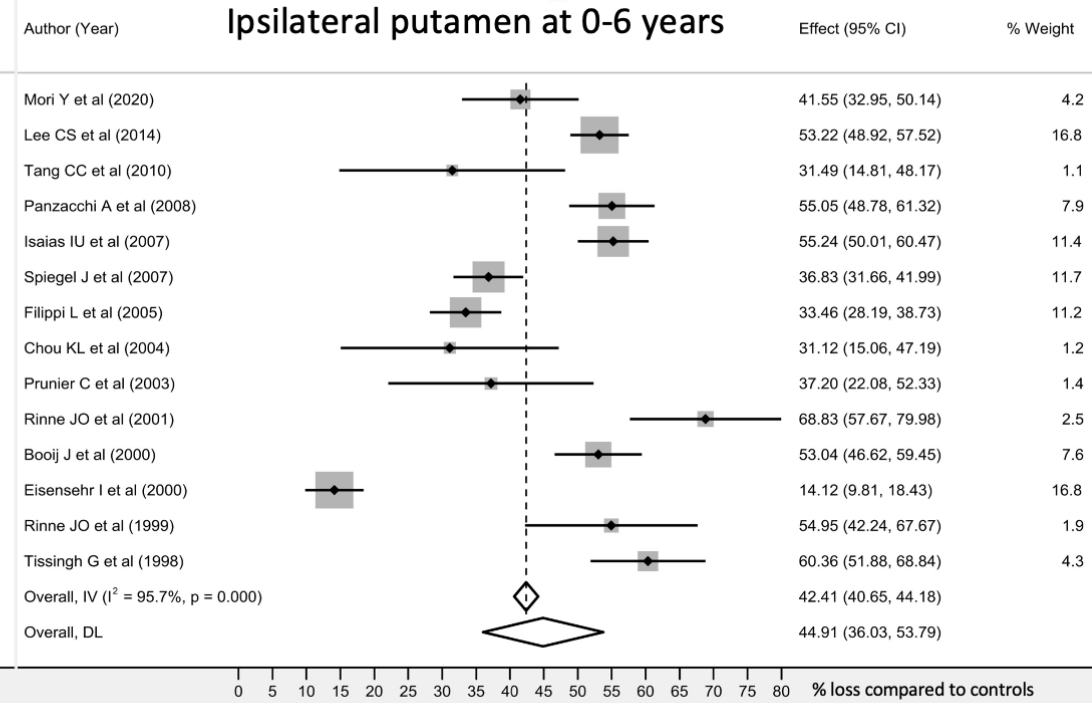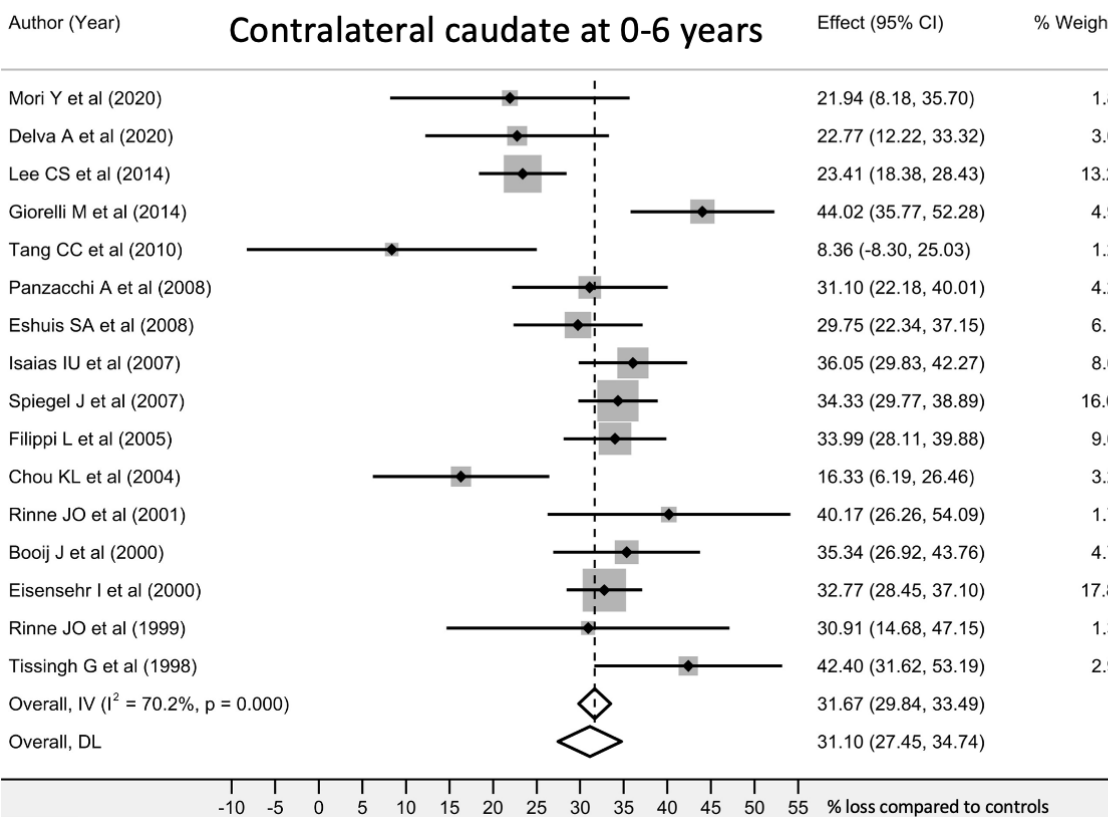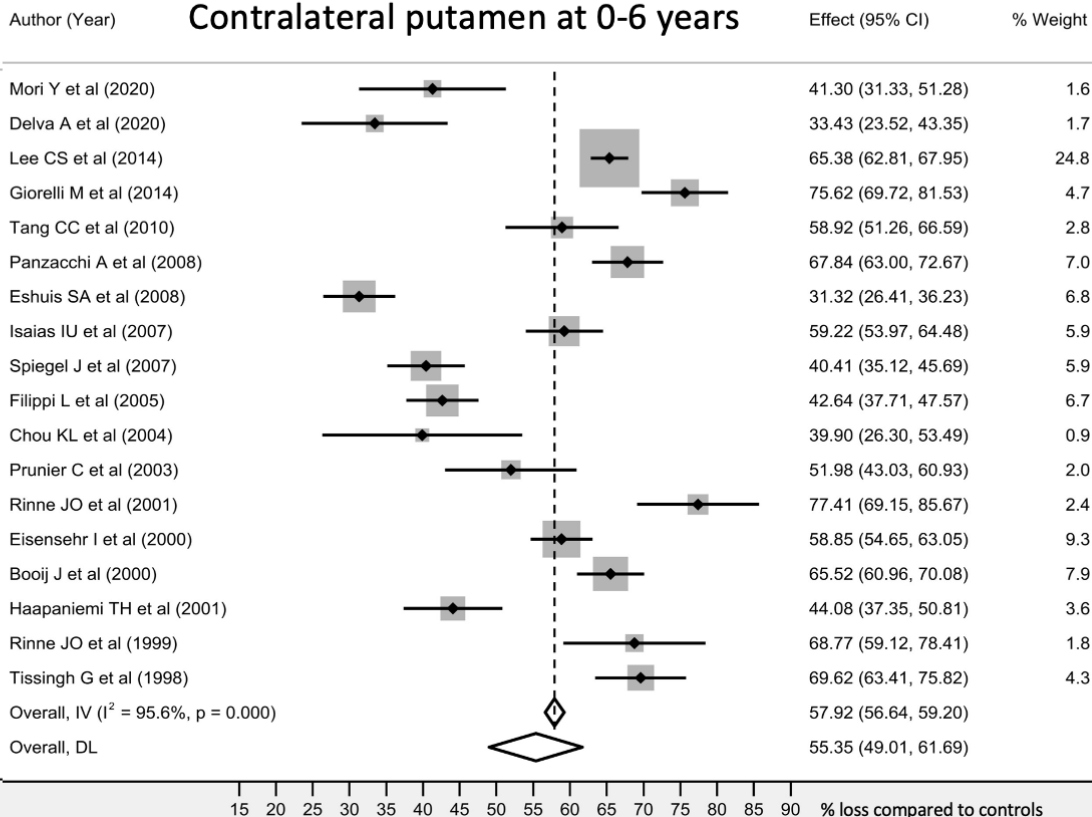

Supplement: Supplementary file 1 — Figure S1. Caudate and putamen dopaminergic activity at 0 to 6 years post‐diagnosis. There was a loss of around 24% in the clinically unaffected caudate, and around 42% in the clinically unaffected putamen. Contralateral losses were around 32% for caudate and 58% for putamen. [file MDC3-10-539-s005.pdf]

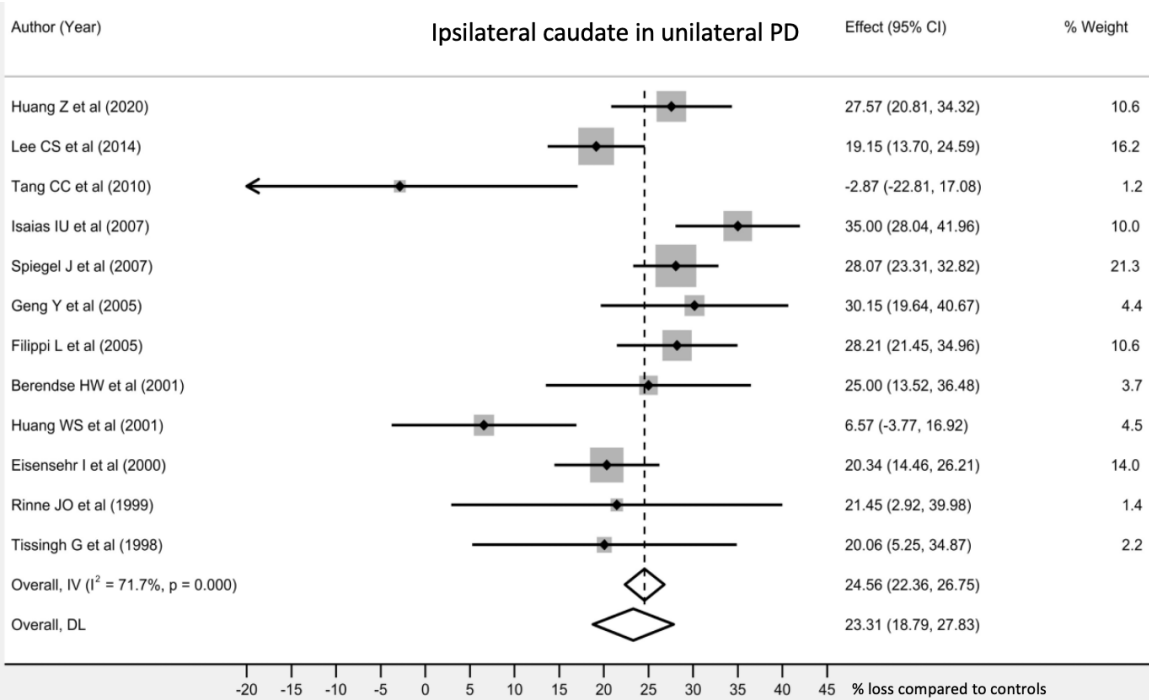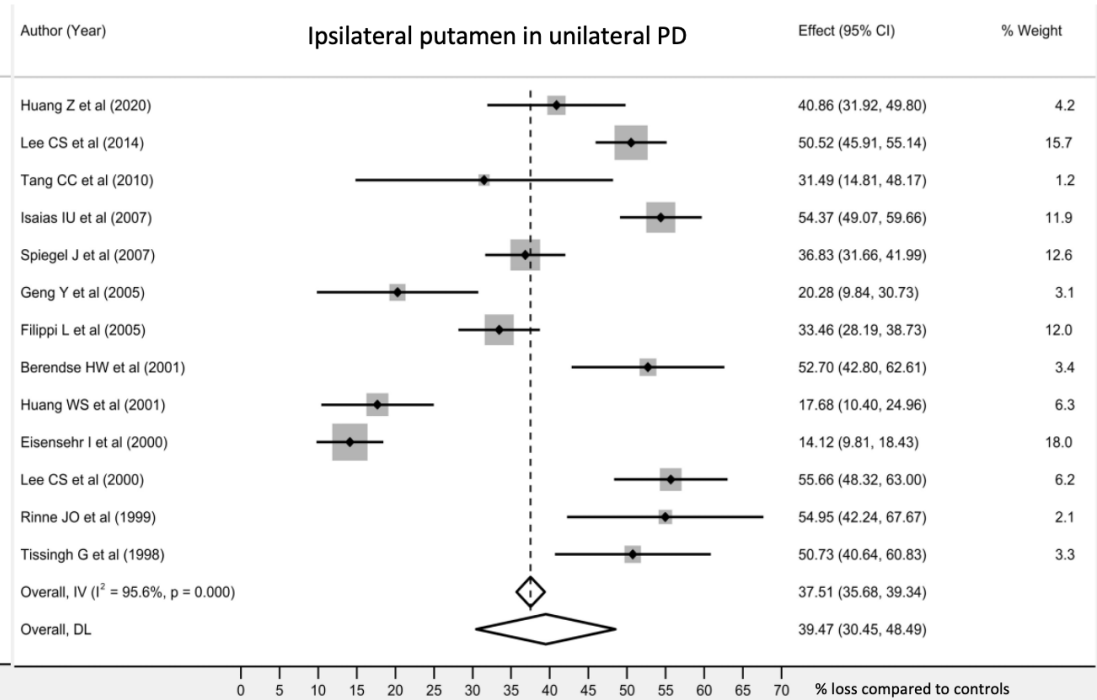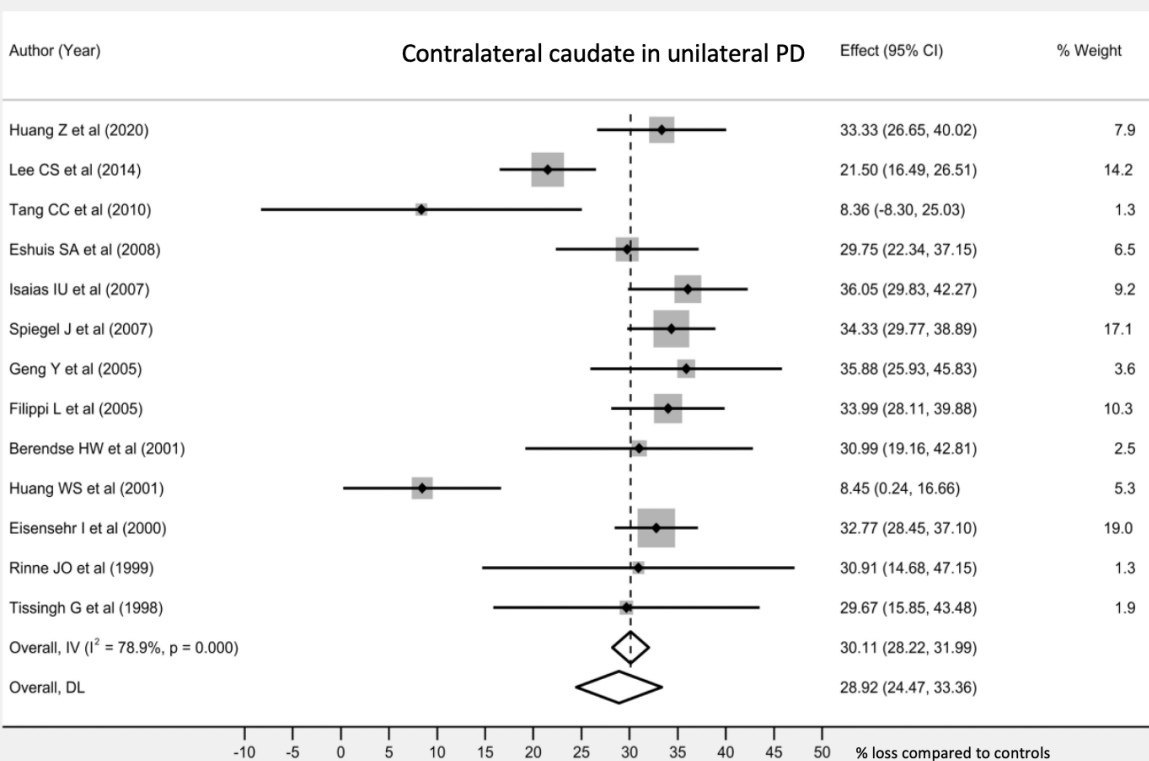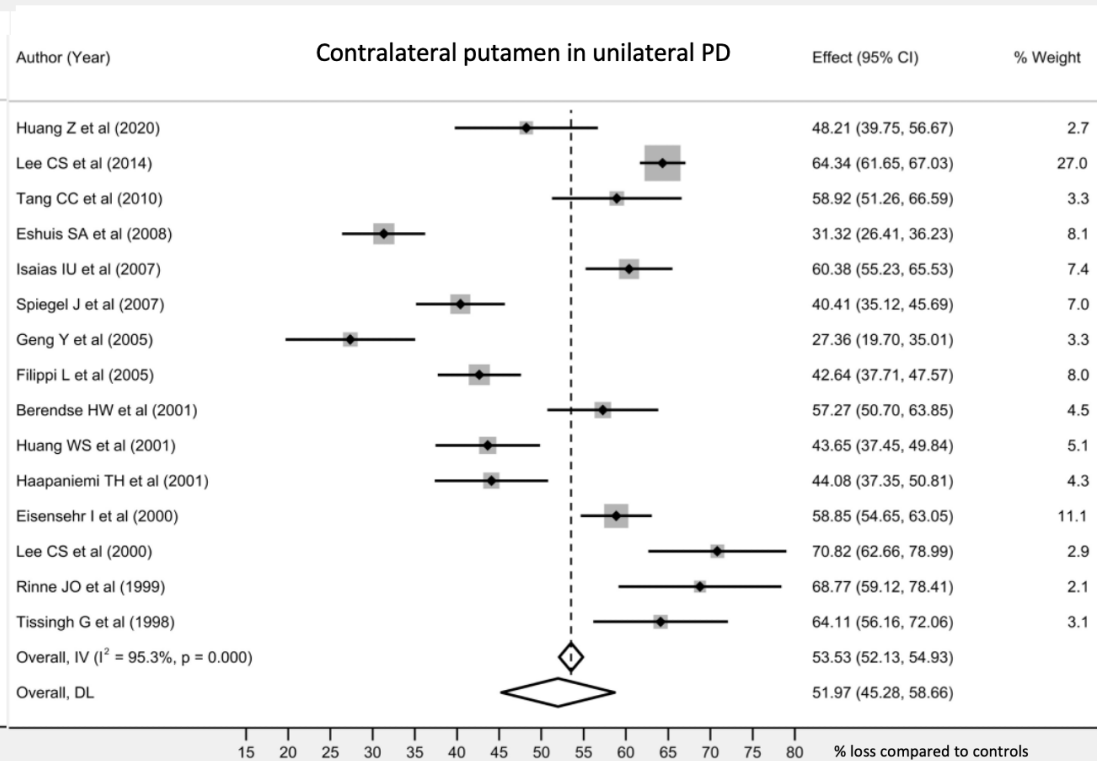

Supplement: Supplementary file 2 — Figure S2. Caudate and putamen dopaminergic activity in early unilateral PD. Loss was around 25% in the clinically unaffected caudate, and around 38% in the clinically unaffected putamen. The contralateral caudate loss was around 30% loss. [file MDC3-10-539-s001.pdf]

# Funnel plot Ipsilateral striatum at 0-6 years

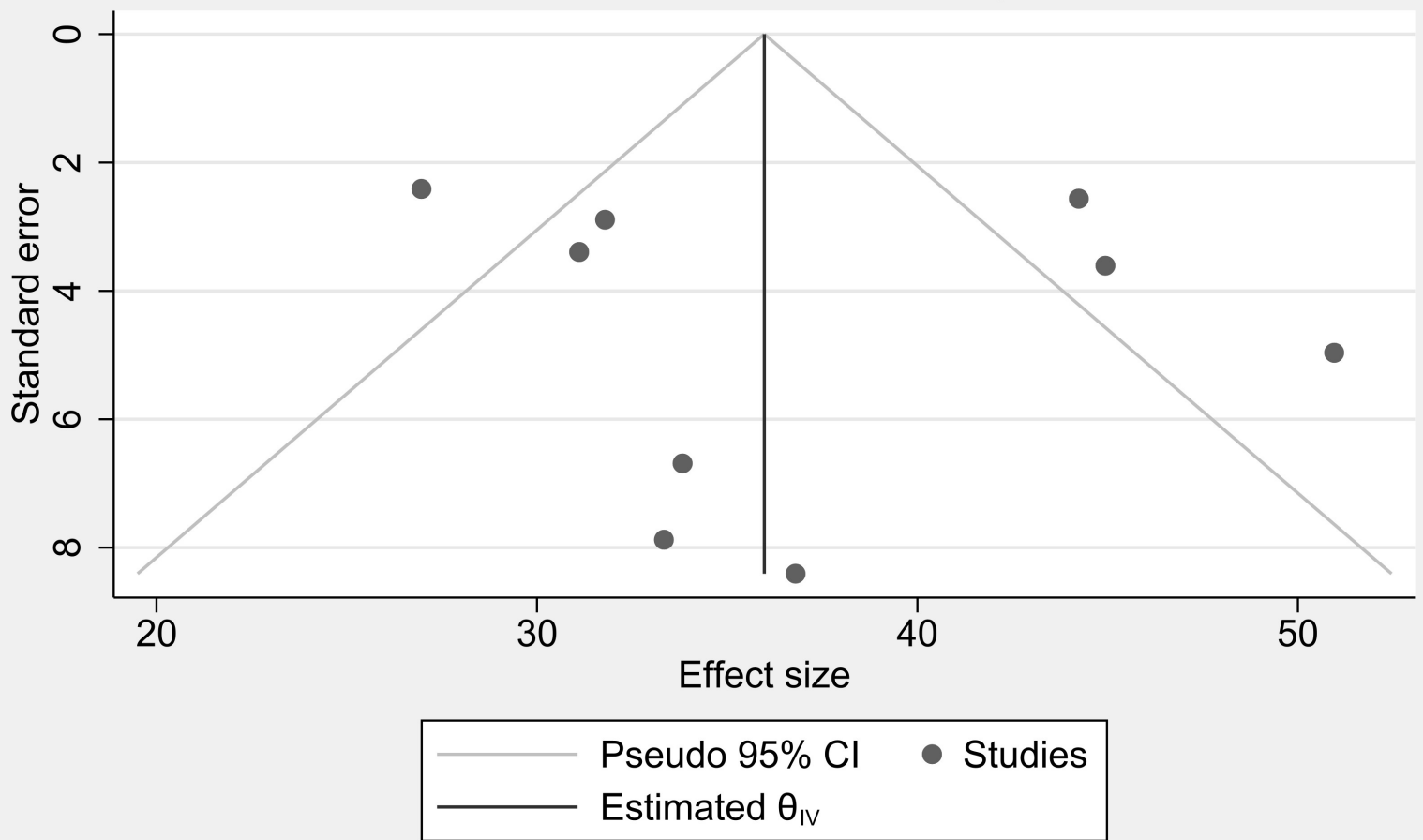

Egger test 1.16 (95% CI: -0.54 to 2.87): p=0.18

Supplement: Supplementary file 3 — Figures S3–S8. Funnel plots of studies at 0–6 years after diagnosis, assessing for publication and selection bias. The largely symmetrical pattern around the midline vertical in study position (each study represented by one dot) showed no evidence of a systematic bias. [file MDC3-10-539-s003.zip › MDC3_13687_Heng et al Supp Fig 3.pdf]

# Funnel plot Contralateral striatum at 0-6 years

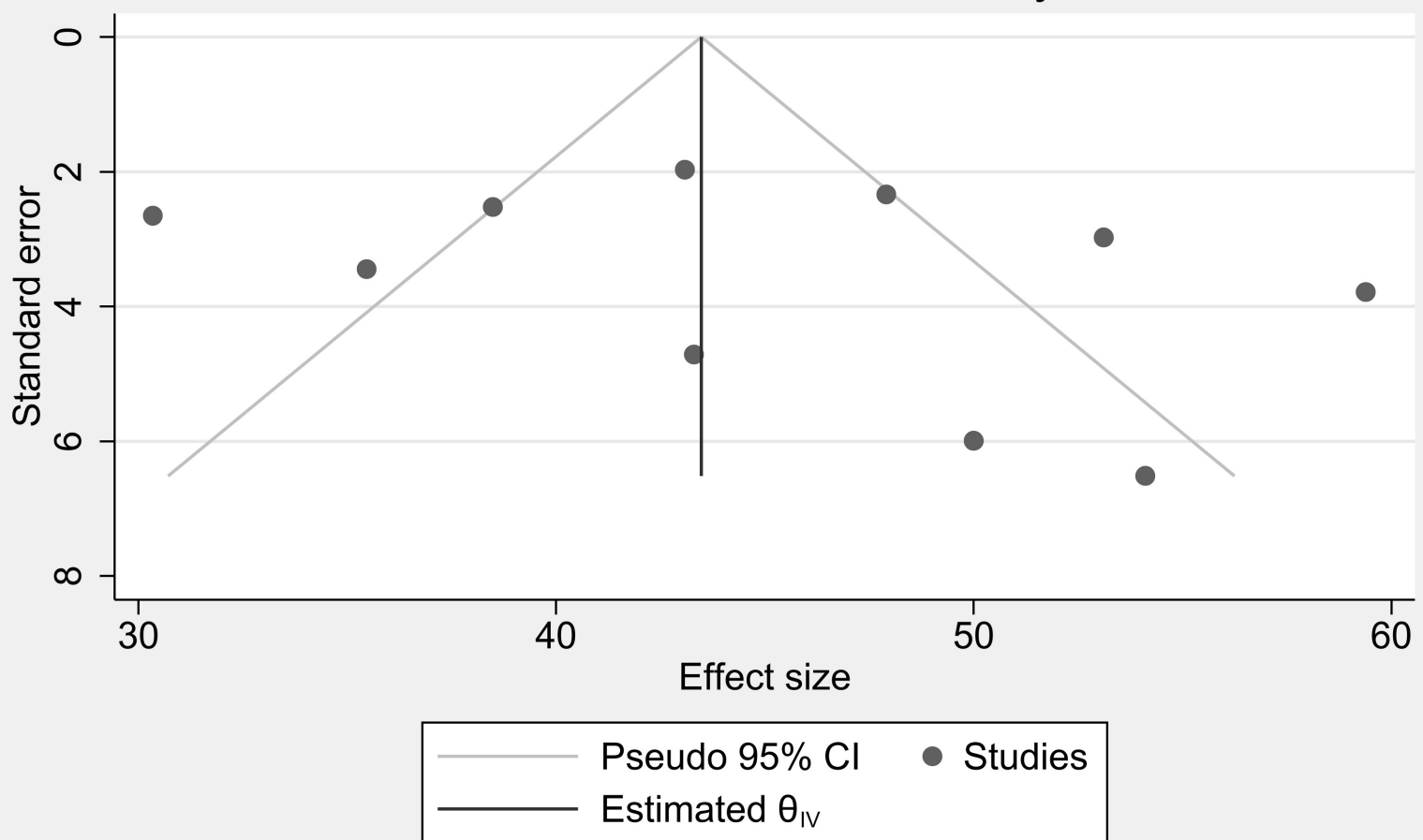

Egger test 2.29 (95% CI: 0.43 to 4.15): p=0.016

Supplement: Supplementary file 3 — Figures S3–S8. Funnel plots of studies at 0–6 years after diagnosis, assessing for publication and selection bias. The largely symmetrical pattern around the midline vertical in study position (each study represented by one dot) showed no evidence of a systematic bias. [file MDC3-10-539-s003.zip › MDC3_13687_Heng et al Supp Fig 4.pdf]

## Funnel plot Ipsilateral caudate at 0-6 years

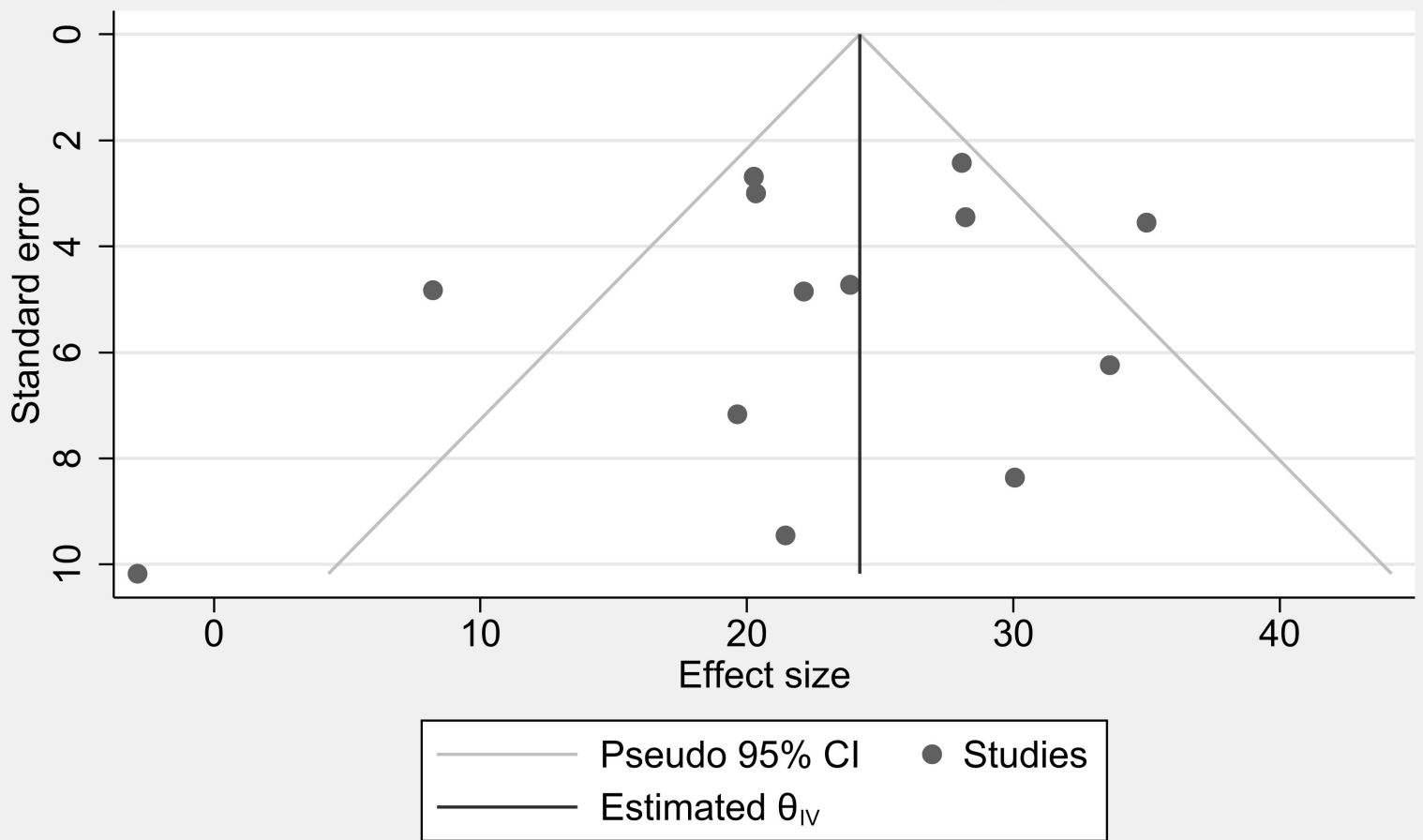

Egger test -1.06 (95% CI: -2.41 to 0.28): p=0.12

Supplement: Supplementary file 3 — Figures S3–S8. Funnel plots of studies at 0–6 years after diagnosis, assessing for publication and selection bias. The largely symmetrical pattern around the midline vertical in study position (each study represented by one dot) showed no evidence of a systematic bias. [file MDC3-10-539-s003.zip › MDC3_13687_Heng et al Supp Fig 5.pdf]

# Funnel plot Ipsilateral putamen at 0-6 years

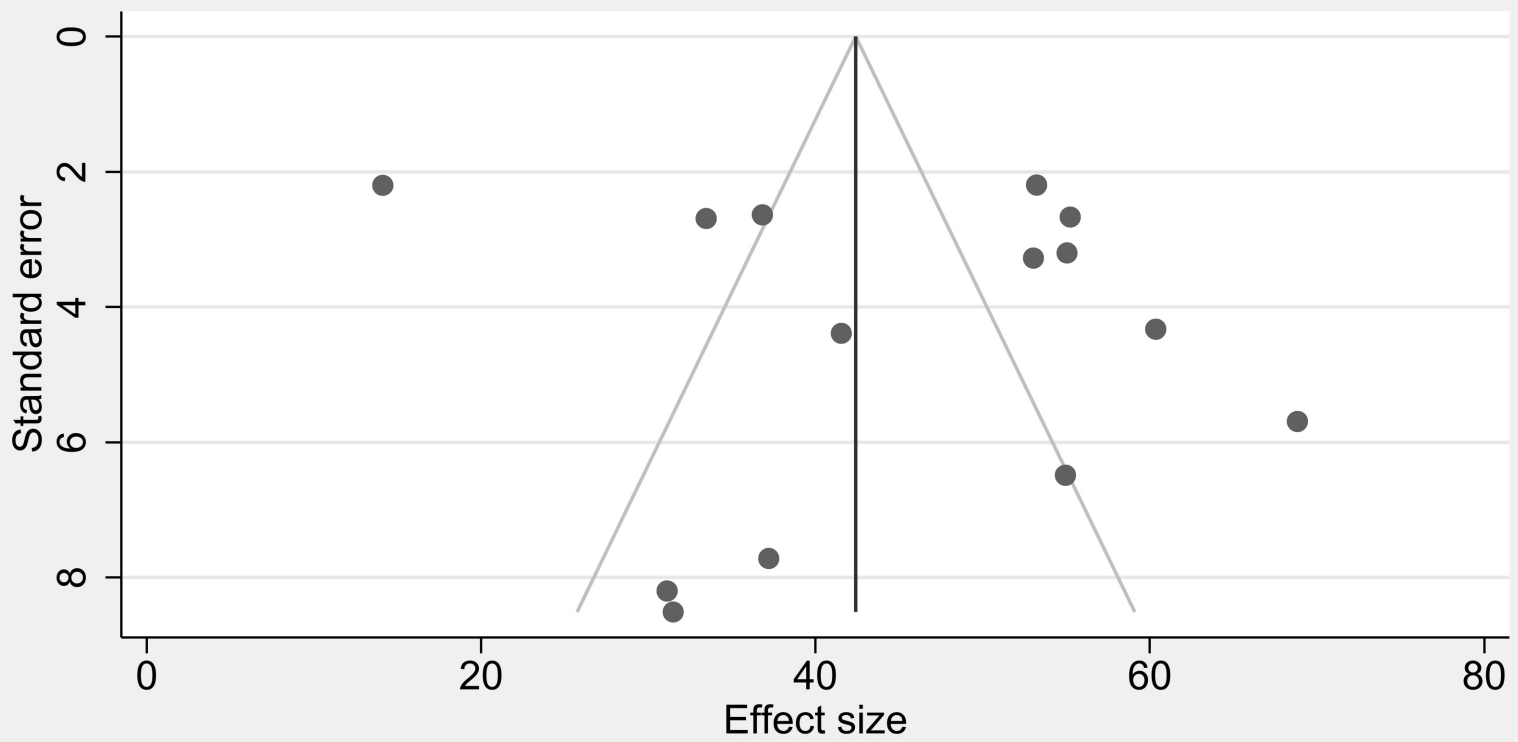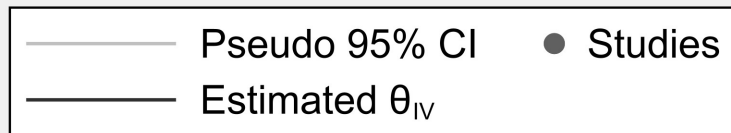

Egger test 2.62 (95% CI: 1.30 to 3.93):  $p < 0.001$

Supplement: Supplementary file 3 — Figures S3–S8. Funnel plots of studies at 0–6 years after diagnosis, assessing for publication and selection bias. The largely symmetrical pattern around the midline vertical in study position (each study represented by one dot) showed no evidence of a systematic bias. [file MDC3-10-539-s003.zip › MDC3_13687_Heng et al Supp Fig 7.pdf]

# Funnel plot

## Contralateral caudate in unilateral PD

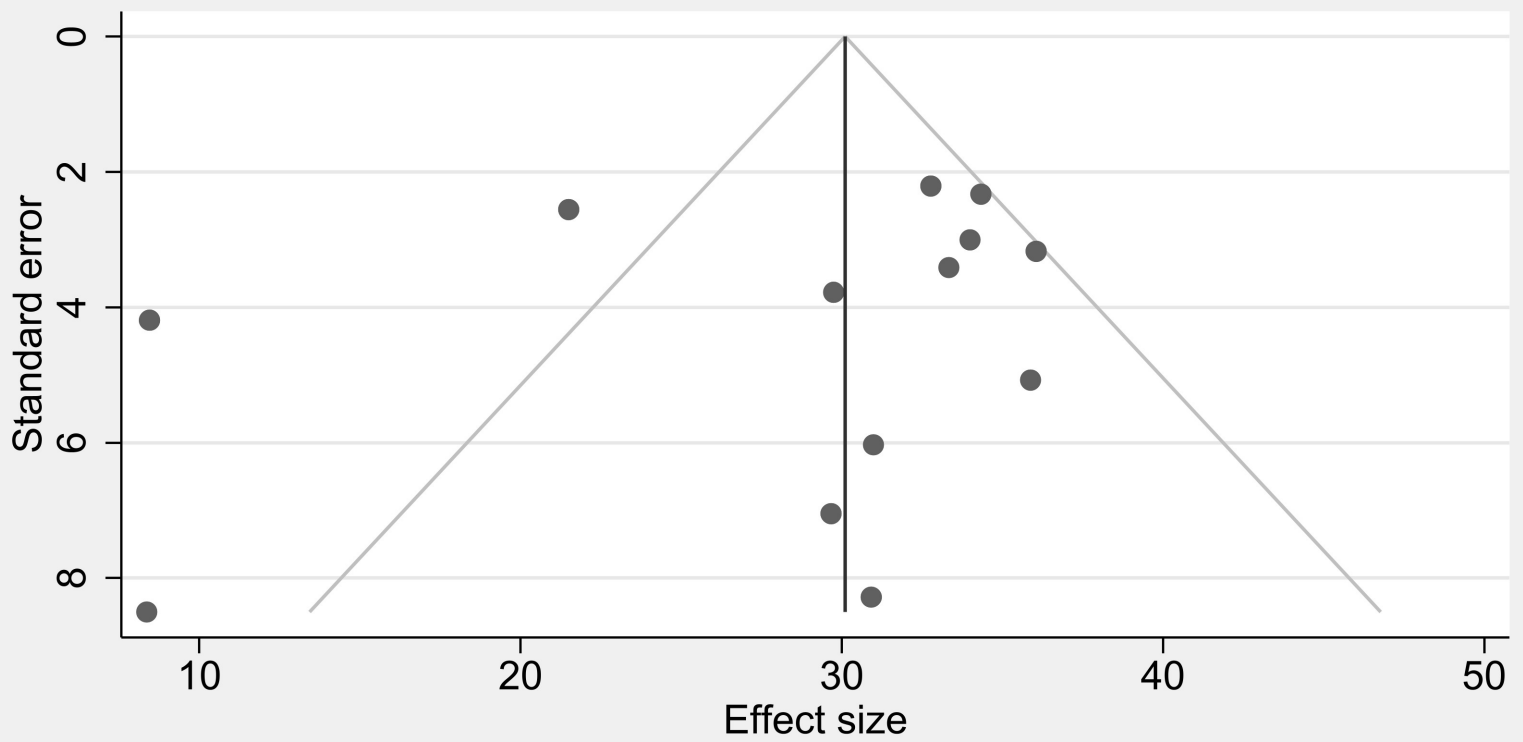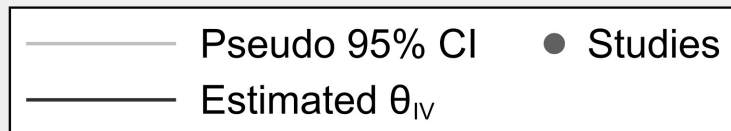

Egger test -1.43 (95% CI: -2.84 to -0.01): p=0.048

Supplement: Supplementary file 4 — Figures S9–S14. Funnel plots of studies of unilateral PD, assessing for publication and selection bias. The largely symmetrical pattern around the midline vertical in study position (each study represented by one dot) showed no evidence of a systematic bias. [file MDC3-10-539-s002.zip › MDC3_13687_Heng et al Supp Fig 12.pdf]
