# Supplementary material for: Striatal Dopamine Loss in Early Parkinson's Disease: Systematic Review and Novel Analysis of Dopamine Transporter Imaging
Source: Mov Disord Clin Pract. 2023 Feb 17;10(4):539–46. doi: 10.1002/mdc3.13687 (PMC10105104; doi:10.1002/mdc3.13687)
Supplement: Supplementary file 3 — Figures S3–S8. Funnel plots of studies at 0–6 years after diagnosis, assessing for publication and selection bias. The largely symmetrical pattern around the midline vertical in study position (each study represented by one dot) showed no evidence of a systematic bias. [file MDC3-10-539-s003.zip › MDC3_13687_Heng et al Supp Fig 6.pdf]

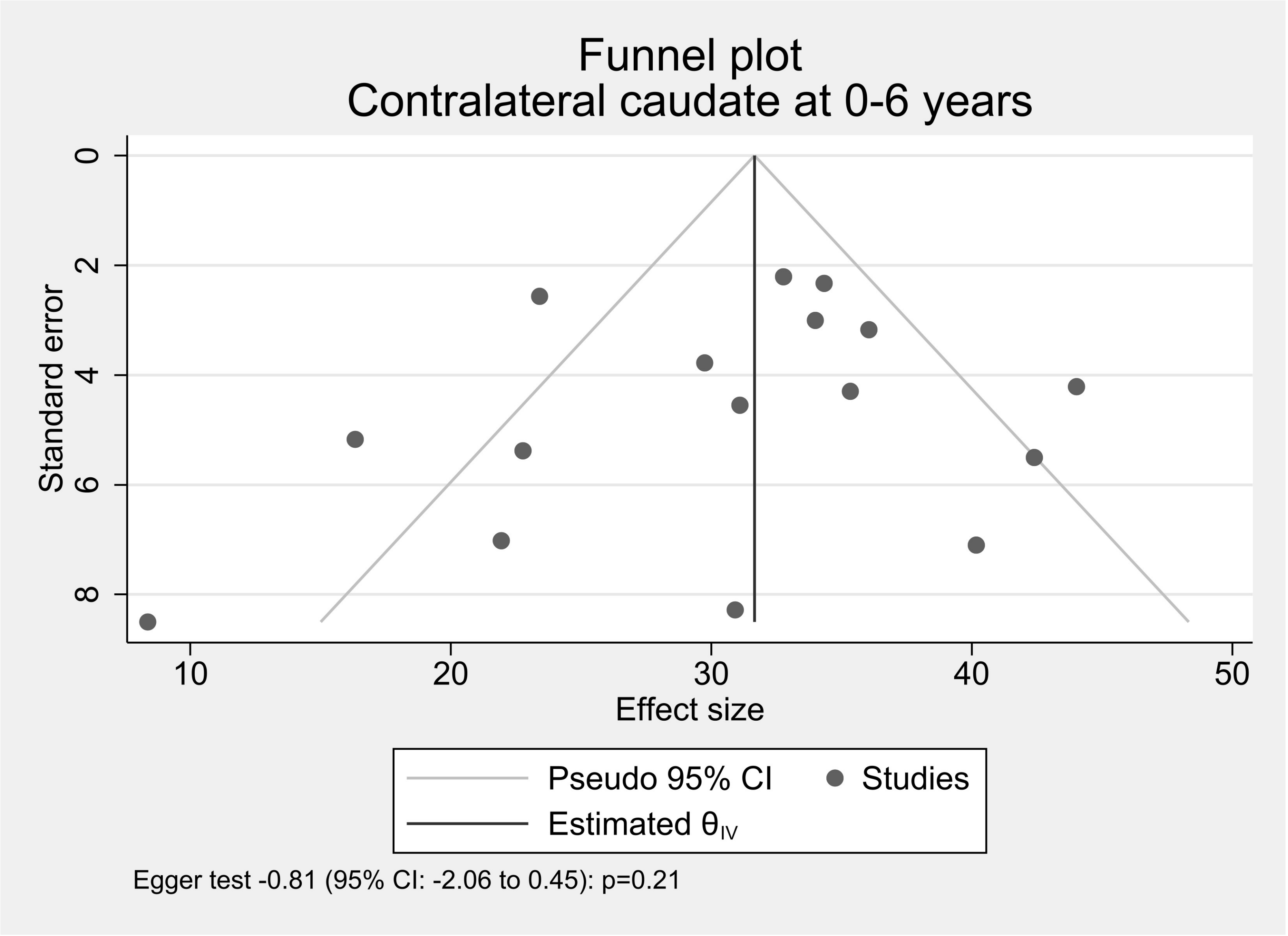
Funnel plot
Contralateral caudate at 0-6 years
Standard error
0
2
4
6
8
10
20
30
40
50
Effect size
Pseudo 95% CI
Studies
Estimated θ_{IV}
Egger test -0.81 (95% CI: -2.06 to 0.45): p=0.21
