# Supplementary material for: Striatal Dopamine Loss in Early Parkinson's Disease: Systematic Review and Novel Analysis of Dopamine Transporter Imaging
Source: Mov Disord Clin Pract. 2023 Feb 17;10(4):539–46. doi: 10.1002/mdc3.13687 (PMC10105104; doi:10.1002/mdc3.13687)
Supplement: Supplementary file 4 — Figures S9–S14. Funnel plots of studies of unilateral PD, assessing for publication and selection bias. The largely symmetrical pattern around the midline vertical in study position (each study represented by one dot) showed no evidence of a systematic bias. [file MDC3-10-539-s002.zip › MDC3_13687_Heng et al Supp Fig 14.pdf]

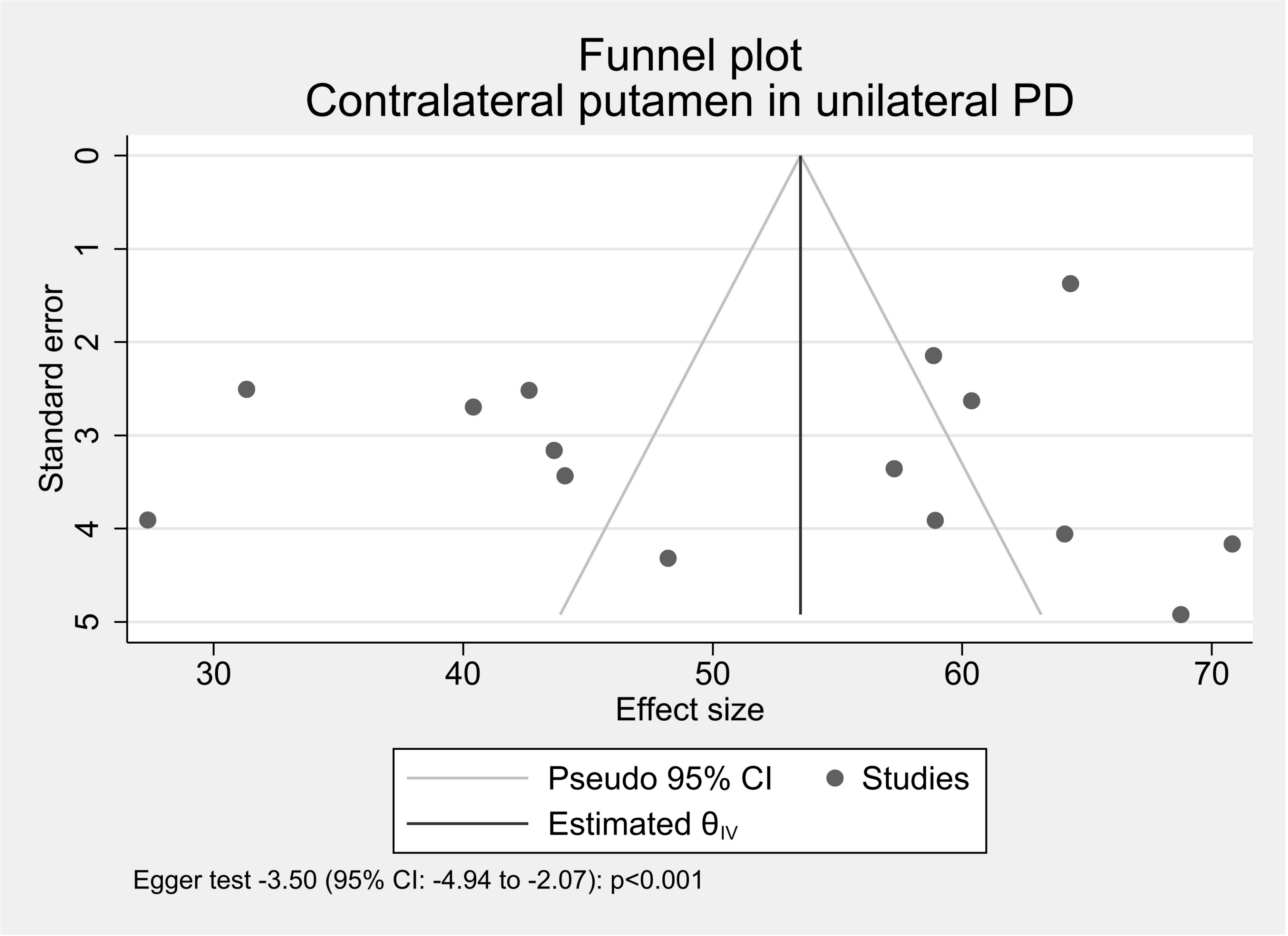

Funnel plot
Contralateral putamen in unilateral PD
Standard error
0
1
2
3
4
5
30
40
50
60
70
Effect size
Pseudo 95% CI
Studies
Estimated θIV
Egger test -3.50 (95% CI: -4.94 to -2.07): p<0.001
