# Supplementary material for: Striatal Dopamine Loss in Early Parkinson's Disease: Systematic Review and Novel Analysis of Dopamine Transporter Imaging
Source: Mov Disord Clin Pract. 2023 Feb 17;10(4):539–46. doi: 10.1002/mdc3.13687 (PMC10105104; doi:10.1002/mdc3.13687)
Supplement: Supplementary file 5 — Table S1. Risk bias analysis of papers based on the QUADAS‐2 tool evaluating the diagnostic use of dopamine transporter imaging in early Parkinson's disease. Table S2. Summary of 27 case control studies evaluating the diagnostic use of dopamine transporter imaging in early Parkinson's disease. Table S3. Meta‐regression for type of tracer used. Reference (baseline) category is an isotope with 123Iodine Table S4. Patient characteristics in the PPMI study. Table S5. Striatal dopamine loss by disease duration and grading in the PPMI study, analyzed by robust standard errors. Table S6. Striatal dopamine loss by disease duration and grading in the PPMI study, analyzed using generalized estimating equations. [file MDC3-10-539-s004.docx]

**Supplementary Table 1.** Risk bias analysis of papers based on the QUADAS-2 tool evaluating the diagnostic use of dopamine transporter imaging in early Parkinson’s disease.

| Author | Patient selection | Index test | | Reference standard | Flow and timing |
| --- | --- | --- | --- | --- | --- |
| Delva A et al 2020 | Unclear | | Unclear | Low | Low |
| Huang Z 2020 | Unclear | | Low | Low | Unclear |
| Mori Y et al 2020 | Unclear | | Unclear | Unclear | Unclear |
| Giorelli M et al 2014 | Unclear | | Unclear | Low | Low |
| Lee CS 2014 | Unclear | | Unclear | Low | Low |
| Tang CC 2010 | Unclear | | Unclear | Low | Unclear |
| Eshuis SA 2009 | Low | | Unclear | Unclear | Low |
| Panzacchi A et al 2008 | Unclear | | Unclear | Low | Low |
| Isaias IU 2007 | Unclear | | Low | Low | Unclear |
| Spiegel J 2007 | Unclear | | Low | Low | Low |
| Spiegel J 2006 | Unclear | | Low | Low | Low |
| Filippi L 2005 | Unclear | | Low | Unclear | Unclear |
| Geng Y 2005 | Unclear | | Unclear | Low | Unclear |
| Wang J 2005 | Unclear | | Low | Low | Unclear |
| Chou KL et al 2004 | Unclear | | High | Low | Low |
| Linke R 2004 | Unclear | | Unclear | High | High |
| Prunier C et al 2003 | Unclear | | Unclear | Low | Low |
| Berendse HW 2001 | Unclear | | Unclear | Unclear | High |
| Haapaniemi TH 2001 | Unclear | | Low | Low | Low |
| Huang WS 2001 | Unclear | | Unclear | Unclear | Unclear |
| Rinne JO et al 2001 | Unclear | | Unclear | Low | Low |
| Booij J et al 2000 | Unclear | | Low | Low | Low |
| Eisensehr I 2000 | Unclear | | Unclear | Low | Unclear |
| Lee CS 2000 | Unclear | | Unclear | Unclear | Unclear |
| Rinne JO 1999 | Unclear | | Unclear | Low | Unclear |
| Tissingh G 1998 | Unclear | | Low | Unclear | Unclear |
| Marek KL 1996 | Low | | Unclear | Low | Low |

**Supplementary Table 2.** Summary of 27 case control studies evaluating the diagnostic use of dopamine transporter imaging in early Parkinson’s disease.

| *Author*  *(Year)* | Isotope | Number  of  cases (controls) | Age in years | Disease duration in  months | Gender  (% male) | H&Y | UPDRS 3 | LEDD in mg/day | Percentage loss of dopamine transporter activity vs controls (95% CI) | | | | | |  |
| --- | --- | --- | --- | --- | --- | --- | --- | --- | --- | --- | --- | --- | --- | --- | --- |
|  |  |  |  |  |  |  |  |  | Striatum | | Caudate | | Putamen | | |
|  |  |  |  |  |  |  |  |  | Contra | Ipsi | Contra | Ipsi | Contra | Ipsi | |
| *Delva A et al (2020) (1)* | ^123^I-FP-CIT | 9 (14) | 64.3 (6.8) | 18.2 (10.6) | 3 (33.3) | 1-2 | 21.8 (10.0) | 225.0 (123.0) | - | - | 22.8 (12.2-33.3) | *-* | 33.4 (23.5-43.4) | ^-^ | |
| *Huang Z et al (2020) (2)* | ^11^C-CFT | 23 (15) | 63.2 (5.5) | 33.6 (26.4) | Not stated | 1 | 15.0  (5.8) | Not stated | - | - | 33.3  (26.7-40.0) | 27.6  (20.8-34.3) | 48.2  (39.8-56.7) | 40.9 (31.9-49.8) | |
| *Mori Y et al (2020) (3)* | ^11^C-CFT | 10 (12) | 57.1 (6.2) | 19.2 (10.1) | 3 (30.0) | 1-2 | 10.2 (2.4) | Drug-naïve | - | - | 21.9  (8.2-35.7) | 19.6 (5.6-33.7) | 41.3 (31.3-51.3) | 41.6 (33.0-50.1) | |
| *Giorelli M et al (2014) (4)* | ^123^I-β-CIT | 31 (22) | 69.1 (8.2) | Less than 2 years | 22 (71.0) | 1-2 | 27.1 (12.0) | Not stated | - | - | 44.0 (35.8-52.3) | - | 75.8 (69.7-81.5) | - | |
| *Lee CS et al (2014) (5)* | ^18^F-FP-CIT | 39 (34) | 56.1 (11.6) | 7.7  (4.3) | 24 (61.5) | 1-2 | Not stated | Drug naïve | - | - | 23.4 (18.4-28.4) | 20.3 (15.0-25.5) | 65.4 (62.8-68.0) | 53.2 (48.9-57.5) | |
|  |  | 31 (34) | 55.8 (10.9) | 7.6  (4.4) | 20 (64.5) | 1 | 4.3  (1.9) | Drug-naïve | - | - | 21.5 (16.5-26.5) | 19.2 (13.7-24.6) | 64.3 (61.7-67.0) | 50.5 (45.9-55.1) | |
| *Tang CC et al (2010) (6)* | ^18^F-FP-CIT | 15 (10) | 58.0 (10.2) | Less than 2 years | 11 (73.3) | 1-1.5 | 9.0  (4.5) | Not stated | - | - | 8.4 (-8.3-25.0) | -2.9 (-22.8-17.1) | 58.9 (51.3-66.6) | 31.5 (14.8-48.2) | |
| *Eshuis SA et al (2009) (7)* | ^123^I-FP-CIT | 11  (10) | 51.3 (10.1) | 1 to 36 | 8 (72.7) | 1-1.5 | Not stated | Not stated | 30.3  (25.2-35.5) | - | 29.8 (22.3-37.2) | - | 31.3 (26.4-36.2) | - | |
| *Panzacchi A et al (2008) (8)* | ^11^C-FECIT | 24 (11) | 53.3 (13.7) | 20.8 (8.0) | Not stated | 1-2 | 16.6 (8.3) | 367.4 (122.1) | - | - | 31.1 (22.2-40.0) | 22.1 (12.6-31.6) | 67.8 (63.0-72.9) | 55.1 (48.8-61.3) | |
| *Isaias IU et al (2007) (9)* | ^123^I-FP-CIT | 20 (31) | 59.5 (10.5) | 18.5 (11.2) | 11 (55.0) | 1 | 17  (7.3) | Drug-naïve | 47.9  (43.3-52.5) | 44.2 (39.2-49.3) | 36.1 (29.8-42.3) | 35.0 (28.0-42.0) | 60.4 (55.2-65.5) | 54.4 (49.1-59.7) | |
| *Spiegel J et al (2007) (10)* | ^123^I-FP-CIT | 67 (19) | 58.9 (10.7) | 24.1 (16.6) | 38 (56.7) | 1 | 11.3  (4.8) | Not stated | - | - | 34.3 (29.8-38.9) | 28.1 (23.3-32.8) | 40.4 (35.1-45.7) | 36.8 (31.7-42.0) | |
| *Spiegel J et al (2006) (11)* | ^123^I-FP-CIT | 32 (19) | 57.0 (9.7) | 25.2 (22.8) | 22 (68.8) | 1 | 11.6  (4.9) | Not stated | 35.5  (28.7-42.2) | 31.1 (24.5-37.8) | - | - | - | - | |
| *Filippi L et al (2005) (12)* | ^123^I-FP-CIT | 29 (18) | 61.3 (10.5) | 25.8 (18.4) | 16 (55.2) | 1 | Not stated | Drug-naïve | 38.5  (33.6-43.4) | 31.8 (26.1-37.5) | 34.0 (28.1-39.9) | 28.2 (21.5-35.0) | 42.6 (37.7-47.6) | 33.5 (28.2-38.7) | |
| *Geng Y et al (2005) (13)* | ^99m^Tc-TRODAT-1 | 24 (18) | 57.1 (10.7) | Not stated | 13 (54.2) | 1 | Not stated | Not stated | 31.2  (23.7-38.7) | 25.7 (17.0-34.4) | 35.9 (25.9-45.8) | 30.2 (19.6-40.7) | 27.4 (19.7-35.0) | 20.3 (9.8-30.7) | |
| *Wang J et al (2005) (14)* | ^99m^Tc-TRODAT-1 | 27 (10) | 54.3 (10.3) | 23.0  (9.8) | 16 (59.3) | 1-1.5 | Not stated | Not stated | 50.0  (38.3-61.7) | 33.3 (17.9-48.8) | - | - | - | - | |
| *Chou KL et al (2004) (15)* | ^99m^Tc-TRODAT-1 | 29 (38) | 59.2 (11.8) | 21.9 (12.4) | 20 (69.0) | 1-2 | 11.9 (5.5) | Not stated | - | - | 16.3 (6.2-26.5) | 8.2 (-1.2-17.7) | 39.9 (28.3-53.5) | 31.1 (15.1-47.2) | |
| *Linke R et al (2004) (16)* | ^123^I-IPT | 29 (23) | 53.3 (9.8) | Not stated | 17 (58.6) | 1 | Not stated | Not stated | 40.0  (35.6-44.4) | 25.0 (20.2-29.8) | - | - | - | - | |
| *Prunier C et al (2003) (17)* | ^123^I-PE2I | 6 (8) | 65.8 (7.4) | 27.2 (21.3) | 4 (66.7) | 1-2 | Not stated | Not stated | 43.3 (34.1-52.5) | 33.8 (20.7-46.9) | - | - | 52.0 (43.0-60.9) | 37.2 (22.1-52.3) | |
| *Berendse HW et al (2001) (18)* | ^123^I-β-CIT | 16 (23) | 58 (6) | Not stated | Not stated | 1-1.5 | Not stated | Not stated | - | - | 31.0 (19.2-42.8) | 25.0 (13.5-36.5) | 57.3 (50.7-63.9) | 52.7 (42.8-62.6) | |
| *Haapaniemi TH et al (2001) (19)* | ^123^I-β-CIT | 19 (21) | 55.4 (11.7) | 17.2 (13.2) | 8 (42.1) | 1-1.5 | 14.8 (7.1) | Drug-naïve | - | - | - | - | 44.1 (37.4-50.8) | - | |
| *Huang WS et al (2001) (20)* | ^99m^Tc-TRODAT-1 | 15 (17) | 62.0 (9.0) | Not stated | 7 (46.7) | 1 | Not stated | Not stated | - | - | 8.5  (0.2-16.7) | 6.6  (-3.8-16.9) | 43.7 (37.5-49.8) | 17.7 (10.4-25.0) | |
| *Rinne JO et al (2001) (21)* | ^18^C-CFT | 8 (5) | 62.8 (10.8) | 19.2 (13.2) | 6 (75.0) | 1.5-2 | 26.9 (9.8) | Drug-naïve | - | - | 40.2 (26.3-54.1) | 30.1 (13.7-46.5) | 77.4 (69.2-85.7) | 68.8 (57.7-80.0) | |
| *Lee CS et al (2000) (22)* | ^11^C-MP | 13 (16) | 57.0 (10.8) | 30.5 (21.6) | 7 (53.8) | 1 | Not stated | Not stated | - | - | - | - | 70.8 (62.7-79.0) | 55.7 (48.3-63.0) | |
| *Booij J et al (2000) (23)* | ^123^I-FP-CIT | 32 (36) | 53.0 (11.3) | 24.0 (15.6) | 24 (75.0) | 1-2.5 | 15.0 (7.6) | Drug-naïve | 53.1 (47.3-58.9) | 44.9 (37.9-52.0) | 35.3 (26.9-43.8) | 23.9 (14.6-33.1) | 65.5 (61.0-70.1) | 53.0 (46.6-59.5) | |
| *Eisensehr I et al (2000) (24)* | ^123^I-IPT | 14 (14) | 50.3 (9.9) | 20.0 (11.0) | 10 (71.4) | 1 | Not stated | Drug-naïve | 43.1  (39.2-46.9) | 27.0 (22.2-31.7) | 32.8 (28.5-37.1) | 20.3 (14.5-26.2) | 58.9 (54.7-63.1) | 14.1 (9.8-18.4) | |
| *Rinne JO et al (1999) (25)* | ^18^F-β-CIT | 9 (4) | 60.0 (11.4) | 21.6  (7.2) | 8 (72.7) | 1 | 6.7  (3.1) | Not stated | - | - | 30.9 (14.7-47.2) | 21.5  (2.9-40.0) | 68.8 (59.1-78.4) | 55.0 (42.2-67.7) | |
| *Tissingh G et al (1998) (26)* | ^123^I-β-CIT | 16 (10) | 51.6 (9.5) | 32.6 (18.2) | 8 (50.0) | 1-2.5 | 16.3 (5.6) | Drug-naïve | 59.4 (52.0-66.8) | 51.0 (41.2-60.7) | 42.4 (31.6-53.2) | 33.6 (21.4-45.9) | 69.6 (63.4-75.8) | 60.4 (51.9-68.8) | |
|  |  | 8 (10) | 49.8 (11.1) | 26.3 (21.5) | 4 (50.0) | 1 | 13.8  (5.23) | Drug-naïve | 50.8  (41.3-66.9) | 39.0 (27.4-50.6) | 29.7 (15.9-43.5) | 20.1  (5.3-34.9) | 64.1 (56.2-72.1) | 50.7 (40.6-60.8) | |
| *Marek KL et al (1996) (27)* | ^123^I-β-CIT | 8 (8) | 61.8 (10.7) | 17.3 (16.8) | 5 (62.5) | 1-1.5 | 14.0  (7.3) | 158.3 (170.2) | 54.1  (38.8-42.4) | 36.8 (20.3-53.3) | - | - | - | - | |

Values are shown as mean (SD) unless otherwise stated, Contra=contralateral, Ipsi=ipsilateral, H&Y=Hoehn and Yahr, UPDRS 3=Unified Parkinson’s disease rating scale Part 3, LEDD=levodopa equivalent daily dose.

**Supplementary Table 3.** Meta-regression for type of tracer used. Reference (baseline) category is an isotope with ^123^Iodine

| **Analysis** | **N with ^123^Iodine** | **N without ^123^Iodine** | **Beta (95% CI)** | **P-value** |
| --- | --- | --- | --- | --- |
| **Unilateral PD** | | | | |
| Contralateral striatum | 8 | 2 | -4.28 (-10.89, 2.32) | p=0.20 |
| Ipsilateral striatum | 7 | 2 | -4.43 (-12.35, 3.48) | p=0.27 |
| Contralateral caudate | 7 | 6 | -9.70 (-13.69, -5.71) | p<0.001 |
| Ipsilateral caudate | 6 | 6 | -6.36 (-10.88, -1.84) | p=0.006 |
| Contralateral putamen | 8 | 7 | 9.68 (6.88, 12.49) | p<0.001 |
| Ipsilateral putamen | 6 | 7 | 7.67 (3.92, 11.43) | p<0.001 |
| **0-6 years** | | | | |
| Contralateral striatum | 9 | 1 | 6.69 (-5.21, 18.58) | p=0.27 |
| Ipsilateral striatum | 8 | 1 | -2.71 (-18.33, 12.92) | p=0.73 |
| Contralateral caudate | 9 | 7 | -9.86 (-13.99, -5.73) | p<0.001 |
| Ipsilateral caudate | 6 | 7 | -9.00 (-13.61, -4.39) | p<0.001 |
| Contralateral putamen | 11 | 7 | 11.55 (8.95, 14.14) | p<0.001 |
| Ipsilateral putamen | 7 | 7 | 14.93 (11.25, 18.61) | p<0.001 |

**Supplementary Table 4.** Patient characteristics in the PPMI study.

|  |  | Number of patients | Number of scans | Age at scan, years (SD) | Male % | Disease duration, years (SD) | UPDRS 3 (SD) | On treatment % |
| --- | --- | --- | --- | --- | --- | --- | --- | --- |
| Disease duration  in years | 0-1 | 354 | 364 | 61.8 (9.8) | 65.7 | 0.4 (0.2) | 21.0 (9.1) | 1.9 |
|  | 1-2 | 344 | 352 | 62.8 (9.7) | 65.6 | 1.4 (0.2) | 23.6 (10.2) | 55.7 |
|  | 2-3 | 337 | 350 | 64.1 (9.6) | 66.0 | 2.4 (0.3) | 25.4 (11.3) | 79.1 |
|  | 3-4 | 58 | 62 | 65.8 (10.3) | 62.9 | 3.5 (0.3) | 28.6 (12.1) | 75.8 |
|  | 4-5 | 256 | 257 | 65.8 (9.8) | 63.4 | 4.4 (0.2) | 28.9 (11.4) | 96.1 |
|  | 5+ | 48 | 50 | 66.6 (9.6) | 60.0 | 5.8 (0.6) | 30.6 (15.9) | 92.0 |
|  | 0-6 | 413 | 1421 | 63.7 (9.8) | 65.2 | 2.1 (1.5) | 24.7 (11.0) | 56.9 |
| Hoehn and Yahr | 1 | 7 | 9 | 58.7 (11.3) | 22.2 | 1.9 (1.1) | 8.4 (3.9) | 88.9 |
|  | 1.5 | 157 | 270 | 60.1 (9.8) | 56.3 | 1.7 (1.4) | 14.6 (5.9) | 38.1 |
|  | 2 | 375 | 985 | 63.9 (9.7) | 69.1 | 2.2 (1.6) | 26.0 (9.7) | 59.9 |
|  | 2.5 | 73 | 100 | 67.1 (8.6) | 62.0 | 2.5 (1.7) | 30.1 (10.5) | 68.0 |
|  | 3+ | 42 | 58 | 70.4 (8.5) | 53.4 | 3.3 (1.6) | 44.2 (11.4) | 81.0 |
| <1 year, drug-naïve | 1 and 1.5 | 95 | 95 | 58.2 (10.4) | 60.0 | 0.4 (0.2) | 13.0 (4.4) | 0.0 |

SD=standard deviation, UDPRS 3=Movement Disorder Society Unified Parkinson’s Disease rating scale Part 3.

**Supplementary Table 5.** Striatal dopamine loss by disease duration and grading in the PPMI study, analyzed by robust standard errors.

|  |  | Number of patients | Number of scans | Striatum | Ipsi  striatum | Contra striatum | Ipsi  caudate | Contra caudate | Ipsi putamen | Contra putamen |
| --- | --- | --- | --- | --- | --- | --- | --- | --- | --- | --- |
| Disease duration in years | 0-1 | 354 | 364 | 45.3 (43.0, 47.6) | 39.5 (36.9, 42.1) | 51.2 (49.1, 53.3) | 28.1 (25.2, 31.0) | 39.2 (36.6, 41.7) | 55.3  (53.0, 57.7) | 67.8 (66.1, 69.5) |
|  | 1-2 | 344 | 352 | 51.2 (49.1, 53.3) | 46.2 (43.8, 48.5) | 56.2 (54.2, 58.1) | 34.4 (31.7, 37.2) | 44.7 (42.3, 47.1) | 62.5 (60.4, 64.5) | 72.1 (70.6, 73.6) |
|  | 2-3 | 337 | 350 | 54.2 (52.0, 56.3) | 49.7 (47.3, 52.1) | 58.7 (56.7, 60.6) | 38.4 (35.6, 41.2) | 48.1 (45.6, 50.5) | 65.4 (63.4, 67.4) | 73.4 (72.0, 74.9) |
|  | 3-4 | 58 | 62 | 55.1 (51.0, 59.1) | 51.0 (46.7, 55.4) | 59.1 (55.2, 63.1) | 38.8 (33.7, 44.0) | 47.9 (42.7, 53.0) | 68.0 (64.2, 71.8) | 74.8 (71.9, 77.7) |
|  | 4-5 | 256 | 257 | 60.0 (57.9, 62.0) | 56.1 (53.8, 58.4) | 63.8 (61.9, 65.7) | 45.3 (42.5, 48.0) | 54.5 (52.1, 56.9) | 71.1 (69.4, 72.9) | 76.7 (75.3, 78.2) |
|  | 5+ | 48 | 50 | 60.9 (56.8, 65.0) | 56.8 (52.4, 61.3) | 65.0 (61.0, 68.9) | 46.0 (40.3, 51.7) | 55.5 (50.2, 60.8) | 71.9 (68.6, 75.2) | 78.2 (75.7, 80.7) |
|  | 0-6 | 413 | 1421 | 52.5 (50.5, 54.4) | 47.6 (45.4, 49.8) | 57.3 (55.5, 59.1) | 36.3 (33.7, 38.8) | 46.4 (44.1, 48.6) | 63.4 (61.5, 65.2) | 72.5 (71.2, 73.9) |
| Hoehn and Yahr | 1 | 7 | 9 | 41.6  (31.3, 51.9) | 36.2 (26.9, 45.5) | 47.0 (34.7, 59.2) | 23.3 (13.3, 33.3) | 34.4 (20.8, 48.0) | 54.1 (44.0, 64.3) | 64.4 (52.6, 76.2) |
|  | 1.5 | 157 | 270 | 46.3 (43.3, 49.2) | 39.7 (36.4, 43.1) | 52.8 (50.0, 55.5) | 28.1 (24.3, 32.0) | 40.8 (37.3, 44.3) | 55.9 (52.9, 58.9) | 69.3 (67.4, 71.3) |
|  | 2 | 375 | 985 | 53.3 (51.3, 55.3) | 48.9 (46.6, 51.1) | 57.7 (55.9, 59.6) | 37.4 (34.8, 40.0) | 46.8 (44.5, 49.1) | 64.7 (62.9, 66.6) | 73.0 (71.6, 74.4) |
|  | 2.5 | 73 | 100 | 57.6 (53.7, 61.5) | 53.6 (49.0, 58.2) | 61.6 (58.2, 65.1) | 43.0 (37.2, 48.7) | 52.1 (47.4, 56.8) | 68.3 (65.0, 71.5) | 74.8 (72.6, 77.0) |
|  | 3+ | 42 | 58 | 60.4 (55.8, 65.0) | 56.2 (51.1, 61.2) | 64.7 (60.2, 69.2) | 45.9 (39.9, 51.8) | 56.3 (50.9, 61.8) | 70.4 (66.1, 74.8) | 76.3 (71.9, 80.7) |
| <1 year, drug-naïve | 1 and 1.5 | 95 | 95 | 40.5 (36.9, 44.2) | 33.7 (29.5, 37.9) | 47.4 (44.0, 50.8) | 22.6 (18.1, 27.2) | 34.5 (30.3, 38.7) | 49.1 (45.0, 53.2) | 65.3 (62.6, 67.9) |

Contra=contralateral, Ipsi=ipsilateral. Data are mean (95% confidence intervals).

**Supplementary Table 6.** Striatal dopamine loss by disease duration and grading in the PPMI study, analyzed using generalized estimating equations.

|  |  | Striatum | Ipsi striatum | Contra striatum | Ipsi  caudate | Contra caudate | Ipsi  putamen | Contra putamen |
| --- | --- | --- | --- | --- | --- | --- | --- | --- |
| Disease duration  in years | 0-1 | 45.3 (43.0, 47.6) | 39.5 (36.9, 42.1) | 51.2 (49.1, 53.3) | 28.1 (25.2, 31.0) | 39.2 (36.6, 41.7) | 55.3  (53.0, 57.7) | 67.8 (66.1, 69.5) |
|  | 1-2 | 51.2 (49.1, 53.3) | 46.2 (43.8, 48.5) | 56.2 (54.2, 58.1) | 34.4 (31.7, 37.2) | 44.7 (42.3, 47.1) | 62.5 (60.4, 64.5) | 72.1 (70.6, 73.6) |
|  | 2-3 | 54.2 (52.1, 56.3) | 49.7 (47.4, 52.0) | 58.7 (56.8, 60.6) | 38.4 (35.6, 41.1) | 48.1 (45.7, 50.4) | 65.4 (63.4, 67.3) | 73.4 (72.0, 74.9) |
|  | 3-4 | 55.1 (51.1, 59.1) | 51.0 (46.7, 55.4) | 59.1 (55.2, 63.0) | 38.8 (33.6, 44.0) | 47.9 (42.8, 52.9) | 68.0 (64.3, 71.7) | 74.8 (71.9, 77.6) |
|  | 4-5 | 60.0 (57.9, 62.0) | 56.1 (53.8, 58.4) | 63.8 (61.9, 65.7) | 45.3 (42.5, 48.0) | 54.5 (52.1, 56.9) | 71.1 (69.4, 72.9) | 76.7 (75.3, 78.2) |
|  | 5+ | 60.9 (56.9, 64.9) | 56.8 (52.5, 61.2) | 65.0 (61.1, 68.8) | 46.0 (40.4, 51.6) | 55.5 (50.3, 60.7) | 71.9 (68.7, 75.1) | 78.2 (75.7, 80.6) |
|  | 0-6 | 52.5 (50.5, 54.4) | 47.6 (45.4, 49.8) | 57.3 (55.5, 59.1) | 36.3 (33.7, 38.8) | 46.4 (44.2, 48.6) | 63.4 (61.5, 65.2) | 72.5 (71.2, 73.9) |
| Hoehn and Yahr | 1 | 41.6  (32.3, 50.8) | 36.2 (28.1, 44.3) | 47.0 (35.7, 58.2) | 23.3 (14.9, 31.7) | 34.4 (22.4, 46.3) | 54.1 (44.7, 63.5) | 64.4 (53.0, 75.9) |
|  | 1.5 | 46.3 (43.4, 49.1) | 39.7 (36.5, 43.0) | 52.8 (50.1, 55.4) | 28.1 (24.6, 31.7) | 40.8 (37.6, 44.0) | 55.9 (52.9, 58.9) | 69.3 (67.3, 71.4) |
|  | 2 | 53.3 (51.3, 55.3) | 48.9 (46.7, 51.1) | 57.7 (55.9, 59.6) | 37.4 (34.9, 40.0) | 46.8 (44.5, 49.0) | 64.7 (62.9, 66.6) | 73.0 (71.6, 74.4) |
|  | 2.5 | 57.6 (54.2, 61.0) | 53.6 (49.6, 57.5) | 61.6 (58.5, 64.8) | 43.0 (38.0, 47.9) | 52.1 (47.9, 56.3) | 68.3 (65.4, 71.2) | 74.8 (72.7, 76.9) |
|  | 3+ | 60.4 (56.3, 64.5) | 56.2 (51.1, 60.8) | 64.7 (60.8, 68.6) | 45.9 (40.4, 51.4) | 56.3 (51.3, 61.4) | 70.4 (66.5, 74.4) | 76.3 (72.7, 79.9) |
| <1 year, drug-naïve | 1 and 1.5 | 40.5 (36.9, 44.2) | 33.7 (29.6, 37.8) | 47.4 (44.0, 50.7) | 22.6 (18.1, 27.2) | 34.5 (30.3, 38.7) | 49.1 (45.0, 53.1) | 65.3 (62.6, 67.9) |

Contra=contralateral, Ipsi=ipsilateral. Data are mean (95% confidence interval)

**References**

1. Delva A, Weehaeeghe D, Aalst J, et al. Quantification and discriminative power of 18 F-FE-PE2I PET in patients with Parkinson's disease. Eur J Nucl Med Mol Imaging. 2020; 47(8): 1913-1926.
2. Huang Z, Jiang C, Li L, et al. Correlations between dopaminergic dysfunction and abnormal metabolic network activity in REM sleep behavior disorder. J Cereb Blood Flow Metab. 2020; 40(3): 552-562.
3. Mori Y, Yoshikawa E, Futatsubashi M, Ouchi Y. Neural correlates of standing imagery and execution in Parkinsonian patients: The relevance to striatal dopamine dysfunction. PLoS One. 2020; 15(10): e0240998.
4. Giorelli M, Bagnoli J, Consiglio L, et al. Do non-motor symptoms in Parkinson's disease differ from essential tremor before initial diagnosis? A clinical and scintigraphic study. Parkinsonism Relat Disord. 2014; 20(1): 17-21.
5. Lee CS, Kim SJ, Oh SJ, et al. Uneven age effects of [(18)F]FP-CIT binding in the striatum of Parkinson's disease. Ann Nucl Med. 2014; 28(9): 874-9.
6. Tang CC, Poston KL, Dhawan V, Eidelberg D. Abnormalities in metabolic network activity precede the onset of motor symptoms in Parkinson's disease. J Neurosci. 2010; 30(3): 1049-56.
7. Eshuis SA, Jager PL, Maguire PR, Jonkman S, Dierckx RA, Leenders KL. Direct comparison of FP‐CIT SPECT and F‐DOPA PET in patients with Parkinson's disease and healthy controls. Eur J Nucl Med Mol Imaging 2009;36(3):454–462.
8. Panzacchi A, Moresco RM, Garibotto V, et al. A voxel-based PET study of dopamine transporters in Parkinson's disease: relevance of age at onset. Neurobiol Dis. 2008; 31(1): 102-9.
9. Isaias IU, Benti R, Cilia R, et al. [123I]FP-CIT striatal binding in early Parkinson's disease patients with tremor vs. akinetic-rigid onset. Neuroreport. 2007; 18(14): 1499-502.
10. Spiegel J, Hellwig D, Samnick S, et al. Striatal FP-CIT uptake differs in the subtypes of early Parkinson's disease. Neural Transm (Vienna). 2007; 114(3): 331-5.
11. Spiegel J, Hellwig D, Möllers MO, et al. Transcranial sonography and [123I]FP-CIT SPECT disclose complementary aspects of Parkinson's disease. Brain. 2006; 129(Pt 5): 1188-93.
12. Filippi L, Manni C, Pierantozzi M, Brusa L, Danieli R, Stanzione P, Schillaci O. 123I‐FP‐CIT semi‐quantitative SPECT detects preclinical bilateral dopaminergic deficit in early Parkinson's disease with unilateral symptoms. Nucl Med Commun 2005;26(5):421–426.
13. Geng Y, Shi G, Jiang Y, Xu L, Hu X, Shao Y. Investigating the role of 99mTc-TRODAT-1 SPECT imaging in idiopathic Parkinson’s disease. J Zhejiang Univ Sci B. 2005; 6(1): 22-27.
14. Wang J, Jiang YP, Liu XD, et al. 99mTc-TRODAT-1 SPECT study in early Parkinson's disease and essential tremor. Acta Neurol Scand. 2005; 112(6): 380-5.
15. Chou KL, Hurtig HI, Stern MB, et al. Diagnostic accuracy of [99mTc]TRODAT-1 SPECT imaging in early Parkinson's disease. Parkinsonism Relat Disord. 2004; 10(6): 375-9.
16. Linke R, Eisensehr I, Wetter TC, et al. Presynaptic dopaminergic function in patients with restless legs syndrome: are there common features with early Parkinson's disease? Mov Disord. 2004; 19(10): 1158-62.
17. Prunier C, Payoux P, Guilloteau D, et al. Quantification of dopamine transporter by 123I-PE2I SPECT and the noninvasive Logan graphical method in Parkinson's disease. J Nucl Med. 2003; 44(5): 663-70.
18. Berendse HW, Booij J, Francot CM, et al. Subclinical dopaminergic dysfunction in asymptomatic Parkinson's disease patients' relatives with a decreased sense of smell. Ann Neurol. 2001; 50(1): 34-41.
19. Haapaniemi TH, Ahonen A, Torniainen P, Sotaniemi  KA, Myllylä VV. [123I]beta-CIT SPECT demonstrates decreased brain dopamine and serotonin transporter levels in untreated parkinsonian patients. Mov Disord. 2001; 16(1): 124-30.
20. Huang WS, Lin SZ, Lin JC, Wey SP, Ting G, Liu RS. Evaluation of early-stage Parkinson's disease with 99mTc-TRODAT-1 imaging. J Nucl Med. 2001; 42(9): 1303-8.
21. Rinne JO, Nurmi E, Ruottinen HM, Bergman J, Eskola O, Solin O. [(18)F]FDOPA and [(18)F]CFT are both sensitive PET markers to detect presynaptic dopaminergic hypofunction in early Parkinson's disease. Synapse 2001;40(3):193–200.
22. Lee CS, Samii A, Sossi V, et al. In vivo positron emission tomographic evidence for compensatory changes in presynaptic dopaminergic nerve terminals in Parkinson's disease. Ann Neurol 2000;47(4):493–503.
23. Booij J, Bergmans P, Winogrodzka A, Speelman JD, Wolters EC. Imaging of dopamine transporters with [123I]FP-CIT SPECT does not suggest a significant effect of age on the symptomatic threshold of disease in Parkinson's disease. Synapse. 2001; 39(2): 101-8.
24. Eisensehr I, Linke R, Noachtar S, Schwarz J, Gildehaus FJ, Tatsch K. Reduced striatal dopamine transporters in idiopathic rapid eye movement sleep behaviour disorder. Comparison with Parkinson's disease and controls. Brain. 2000; 123(Pt 6): 1155-60.
25. Rinne JO, Bergman J, Ruottinen H, et al. Striatal uptake of a novel PET ligand, [18F]beta-CFT, is reduced in early Parkinson's disease. Synapse. 1999; 31(2): 119-24.
26. Tissingh G, Bergmans P, Booij J, Winogrodzka A, van Royen EA, Stoof JC, Wolters EC. Drug‐naive patients with Parkinson's disease in Hoehn and Yahr stages I and II show a bilateral decrease in striatal dopamine transporters as revealed by [123I]beta‐CIT SPECT. J Neurol 1999;245(1):14–20.
27. Marek KL, Seibyl JP, Zoghbi SS, et al. [123I] beta‐CIT/SPECT imaging demonstrates bilateral loss of dopamine transporters in hemi‐Parkinson's disease. Neurology 1996;46(1):231–237.
